# Tobacco Smoke Exposure From Prenatal To Adolescent Periods Drives IBD Pathogenesis: Dynamic DNA Methylation Signatures Across Lifespan Stages

**DOI:** 10.1002/advs.202516704

**Published:** 2026-01-11

**Authors:** Han Zhang, Jianhui Zhao, Jie Chen, Xinyi Ma, Siyun Zhou, Alexandra Noble, Rahul Kalla, Judith Wellens, Kun Liu, Evropi Theodoratou, Jack Satsangi, Xue Li

**Affiliations:** ^1^ Department of Big Data in Health Science The Second Affiliated Hospital School of Public Health Zhejiang University School of Medicine Hangzhou Zhejiang China; ^2^ The Key Laboratory of Intelligent Preventive Medicine of Zhejiang Province Hangzhou Zhejiang China; ^3^ Department of Epidemiology The Shaanxi Provincial Key Laboratory of Environmental Health Hazard Assessment and Protection School of Public Health The Fourth Military Medical University Xi'an Shaanxi China; ^4^ Department of Gastroenterology The Third Xiangya Hospital Central South University Changsha Hunan China; ^5^ Xiangya School of Public Health Central South University Changsha Hunan China; ^6^ Translational Gastroenterology Unit Nuffield Department of Medicine Experimental Medicine Division University of Oxford John Radcliffe Hospital Oxford UK; ^7^ Edinburgh IBD Science Unit Centre For Inflammation Research University of Edinburgh Edinburgh UK; ^8^ Department of Gastroenterology and Hepatology Leuven University Hospital Leuven Belgium; ^9^ Centre for Global Health Usher Institute University of Edinburgh Edinburgh UK

**Keywords:** DNA methylation, early life smoking, inflammatory bowel disease, mendelian randomization study

## Abstract

The association between early life exposure to smoking and the risk of inflammatory bowel disease (IBD) needs to be further verified, and the potential role of DNA methylation in the association is unclear. Through an integrated study design, this study demonstrates that maternal smoking during pregnancy (MSDP) is potentially associated with increased risk of IBD, Crohn's disease (CD), and ulcerative colitis (UC) in offspring. In addition, individuals who started smoking in adolescence have a higher risk of developing CD and IBD. Mechanistically, MSDP‐associated DNA methylation alterations in *ADCY7* (newborn), *AKAP8L* (newborn), *TIGD7* (newborn), and *TNF/LTA* (across life stages) are significantly correlated with increased risk of CD in offspring; MSDP‐induced DNA methylation changes in *PRRT1* (newborn), *AHRR* (across life stages), and *MYO1G* (across life stages) show significant associations with UC risk in offspring. Notably, the alterations of DNA methylation status within *AHRR*, *MYO1G*, and *TNF/LTA* loci associated with smoking exposure are present throughout the life course. Collectively, MSDP may serve as an independent risk factor for IBD in offspring, and active smoking during adolescence may cause increased risk of developing CD and IBD. MSDP may contribute to IBD susceptibility by inducing persistent DNA methylation alterations at multiple developmental stages.

## Graphical abstract

This study illustrates that maternal smoking during pregnancy (MSDP) is an independent risk factor for IBD in offspring, and DNA methylation serves as a key mechanistic pathway connecting early‐life smoking exposure with IBD risk in offspring. That highlights the urgent need for preventive interventions targeting prospective parents and minors to mitigate the rising global incidence of IBD.

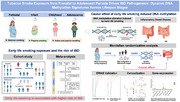

## 1 Introduction

Inflammatory bowel disease (IBD) is a chronic immune‐mediated gastrointestinal disorder comprising two primary subtypes, namely Crohn's disease (CD) and ulcerative colitis (UC). The incidence and prevalence of IBD have been steadily increasing since its emergence in the 20th century, particularly in industrialized nations, [1, 2] posing a significant burden on global healthcare systems. [3, 4] Due to the current absence of curative treatments, increased understanding of risk factors and pathogenic pathways is necessary to advance targeted prevention and treatment strategies in IBD.

The etiology of IBD is influenced by a combination of genetic and environmental factors, with tobacco smoking being one of the most well‐known environmental risk factors investigated. Numerous studies have established a link between smoking status during adulthood and IBD incidence in the elderly [5, 6], which was mediated by epigenetic factors as we have previously shown. [7] Furthermore, early life exposure to smoking, including maternal smoking during pregnancy (MSDP) [8, 9], prenatal smoking exposure [10, 11], and active as well as passive smoking exposure during childhood [12, 13], has also been reported to be associated with increased risk of IBD. However, the heterogeneity among these studies and the limited statistical power have led to inconsistencies and uncertainties regarding the potential associations between early life smoking exposure and the risk of developing IBD. On the other hand, the evidence that supports the association between active smoking before adulthood and the risk of IBD is relatively limited. Lastly, the mechanisms underlying the effect of early life smoking exposure on the development of IBD remain unclear. Epigenetic modifications, particularly DNA methylation, may be key to understanding how environmental factors influence disease pathogenesis. Our previous study demonstrated that DNA methylation alterations within *DNMT3A*, *AHRR*, and *LTA/TNF* loci may mediate the impact of adulthood smoking on IBD incidence in the elderly. [7] However, it remains unclear whether early‐life tobacco smoking exposure promotes the development of IBD by influencing DNA methylation status and whether tobacco smoking exposure at different life stages shares a common biological pathway in the progression of IBD.

Here, we conducted a prospective cohort study to assess the association of passive and personal smoking in early life with the IBD risk at different life stages. Subsequently, an updated meta‐analysis was performed to systematically integrate the available evidence regarding the association between early life smoking exposure and IBD risk. Our secondary objective was to explore whether DNA methylation acts as a mediator for this association and to investigate potential shared epigenetic pathways in the IBD pathogenesis between early life and adult smoke exposure.

## 2 Results

### 2.1 Active Smoking Prior to Adulthood and MSDP are Associated With a Higher Risk of IBD

With a median follow‐up of 13·9 years, 3736 incident IBD, 1086 incident CD, and 2420 incident UC cases were identified in the UK Biobank cohort study. The baseline characteristics of all participants are presented in Table S1. Compared with never exposed to MSDP, people who were exposed to MSDP had an increased risk of IBD (HR = 1·13, 95 %CI: 1·05–1·22), CD (HR = 1·21, 95 %CI: 1·05–1·39), and UC (HR = 1·11, 95 %CI: 1·01–1·23) (Table 1). In addition, individuals who started smoking during adolescence had a higher risk of developing CD (HR = 1·79, 95 %CI: 1·44–2·23) and IBD (HR = 1·35, 95 %CI: 1·18–1·54) (Table 1). While statistically non‐significant, our data suggest a potential protective association between pre‐adulthood active smoking and UC development in individuals aged over 60 years (Table S2). We did not observe any significant association between active smoking during childhood and the risk of IBD and its subtypes. The age‐stratified analysis indicated that the effect of MSDP, as well as active smoking during adolescence, on the risk of CD and IBD mainly emerged in those under 60 years old (Table S2).

**TABLE 1:** The association of maternal smoking during pregnancy and personal active smoking initiation age with the risk of inflammatory bowel disease.

	Overall IBD (cases = 3736)				CD (cases = 1086)				UC (cases = 2420)			
	Model 1		Model 2		Model 1		Model 2		Model 1		Model 2	
	HR (95 %CI)	P	HR (95 %CI)	P	HR (95 %CI)	P	HR (95 %CI)	P	HR (95 %CI)	P	HR (95 %CI)	P
MSDP												
NO	ref		ref		ref		ref		ref		ref	
Yes	1·15 (1·07, 1·24)	2·76E‐04	1·13 (1·05, 1·22)	1·81E‐03	1·22 (1·07, 1·40)	4·14E‐03	1·21 (1·05, 1·36)	7·73E‐03	1·14 (1·03, 1·25)	7·56E‐03	1·11 (1·01, 1·23)	2·71E‐02
Age of personal smoking initiation												
Never smoking	ref		ref		ref		ref		ref		ref	
5–12 years old	1·34 (0·90, 1·99)	1·49E‐01	1·28 (0·86, 1·90)	2·29E‐01	1·48 (0·74, 2·98)	2·72E‐01	1·41 (0·70, 2·84)	3·41E‐01	1·25 (0·75, 2·08)	3·88E‐01	1·19 (0·72, 1·99)	4·97E‐01
13–18 years old	1·41 (1·24, 1·61)	2·19E‐07	1·35 (1·18, 1·54)	1·10E‐05	1·91 (1·54, 2·98)	2·37E‐09	1·79 (1·44, 2·23)	2·06E‐07	1·18 (0·99, 1·40)	6·92E‐02	1·13 (0·95, 1·36)	1·72E‐01
>18 years	1·53 (1·27, 1·84)	7·64E‐06	1·52 (1·26, 1·84)	1·05E‐05	1·98 (1·45, 2·69)	1·47E‐05	1·94 (1·42, 2·65)	3·27E‐05	1·32 (1·03, 1·69)	2·71E‐02	1·33 (1·04, 1·70)	2·52E‐02

IBD, inflammatory bowel disease. CD, Crohn's disease. UC, ulcerative colitis. HR, hazard ratio. 95 %CI, 95 % confidence internal.

Model 1 was adjusted for recruitment age, sex, assessment center, BMI, TDI, aspirin use at baseline, and diabetes and hypertension at baseline, while model 2 was further adjusted for drinking status, personal smoking status (only for MSDP), physical activity, diet score, and the first ten genetic principal components.

### 2.2 Meta‐Analysis Further Verified that Early Life Exposure to Passive Smoking is Potentially Associated With the Risk of CD and UC

**FIGURE 1:**
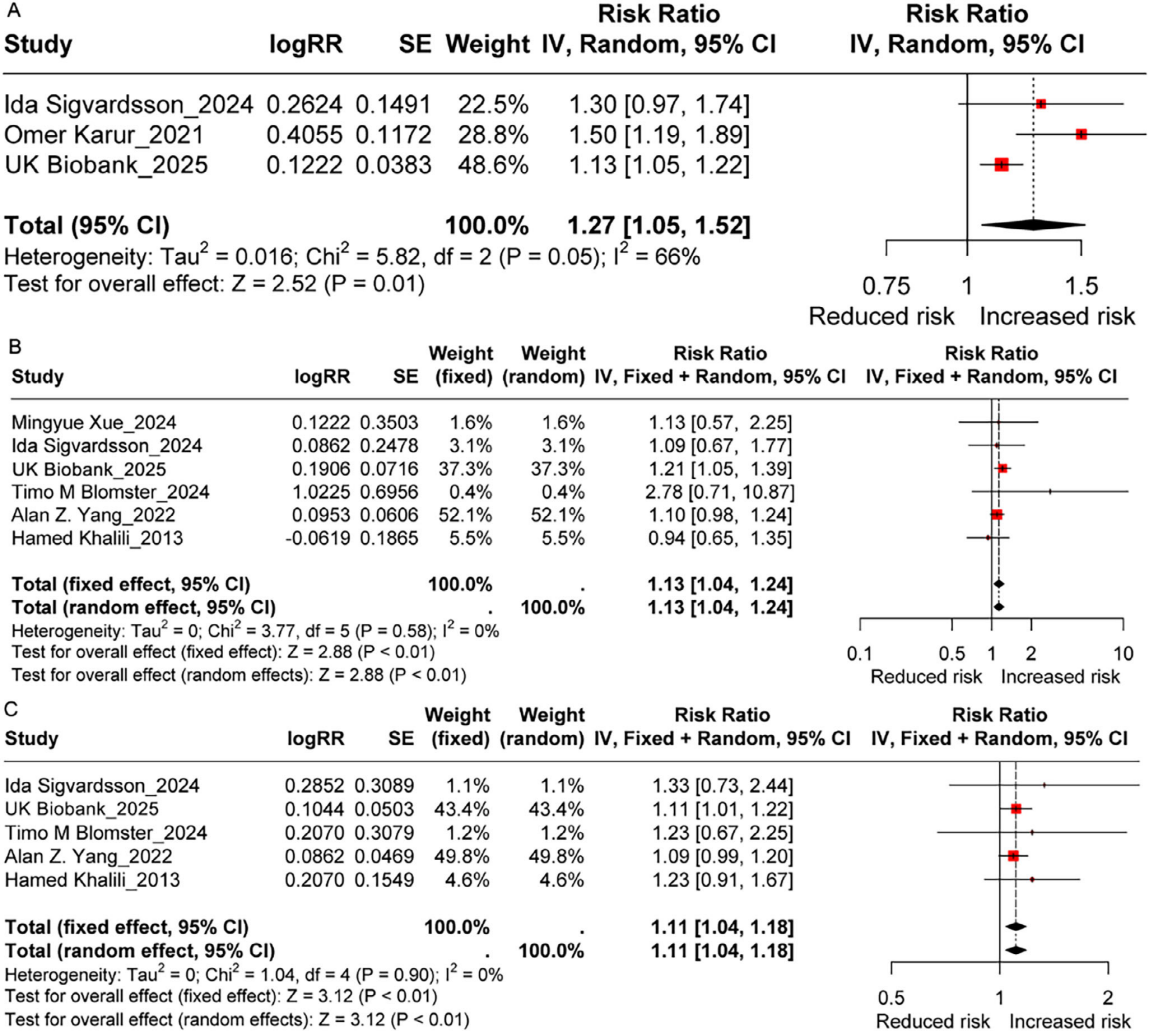
The association between maternal smoking during pregnancy and the risk of inflammatory bowel disease in their offspring. A) inflammatory bowel disease. B) Crohn's disease. C) ulcerative colitis. The sample sizes for the meta‐analyses of inflammatory bowel disease, Crohn's disease, and ulcerative colitis are 853,080, 112,089,2, and 112,185,7, respectively. Meta‐analysis was performed using the R package “metafor”. Data are presented as odds ratios (ORs) with 95 % confidence intervals (CIs). All tests were two‐tailed, and *p*< 0.05 was considered statistically significant.

Seven cohort studies [8, 14, 15, 16, 17, 18] were included in the primary meta‐analysis, contributing to a total sample size of 853,080 for IBD, 112,089,2 for CD, and 112,185,7 for UC, respectively. All included cohort studies were of high quality, with scores exceeding seven (Table S3). The meta‐analysis estimated relative risks of 1·27 (95 %CI: 1·05–1·52) for IBD, 1·13 (95 %CI: 1·04–1·24) for CD, and 1·11 (95 %CI: 1·04–1·18) for UC in the main analysis (Figure 1). Moderate heterogeneity (I^2^ = 66 %) was found in the analysis of overall IBD, and the heterogeneity may be induced by one study (Omer Karur_2021) (Figure 1; Figure S1). For UC outcomes, the Egger's test suggested potential publication bias (P__Egger_ = 0.027), though the small number of studies limits the reliability of this assessment.

In the secondary meta‐analysis, 7 cohort and 16 case‐control studies [10, 11, 12, 13, 19, 20, 21, 22, 23, 24, 25, 26, 27, 28, 29, 30] were included. The quality scores of the included studies ranged from 5 to 8, representing moderate to high quality (Table S3). The association between MSDP and the risk of IBD, CD, and UC remained consistent with the primary analysis when the source of heterogeneity (caused by Sara Aspberg_2006) was excluded (Figures S2–S4). Additionally, exposure to passive smoking during childhood was potentially associated with an increased risk of CD (RR = 1·40, 95 %CI: 1·10–1·78, Figure S5), rather than UC (RR = 0·98, 95 %CI: 0·84–1·13, Figure S6), particularly when the CD occurred in the adult stage (RR = 1·77, 95 %CI: 1·45–2·16, Figure S5). Furthermore, prenatal exposure to tobacco smoking was positively correlated with both CD (RR = 1·55, 95 %CI: 1·13–2·14, Figure S7) and UC (RR = 1·47, 95 %CI: 1·16–1·87, Figure S8) risks, with no heterogeneity or publication bias.

### 2.3 Altered DNA Methylation Caused by Early Life Smoking Exposure May Have an Impact on the Risk of IBD

**FIGURE 2:**
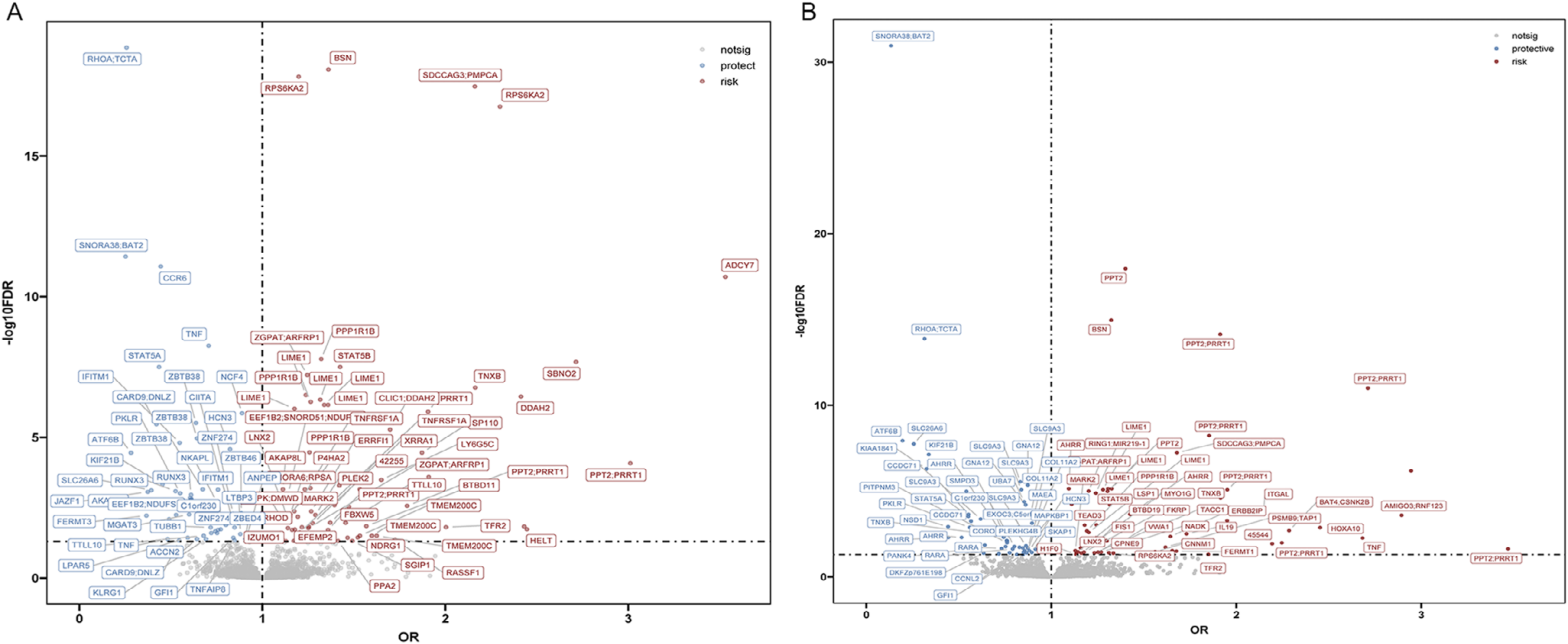
Significant loci in which maternal smoking during pregnancy induced newborn DNA methylation changes are associated with the risk of Crohn's disease and ulcerative colitis in offspring. A) Crohn's disease. B) ulcerative colitis. The plot shows the significant loci associated with the risk of inflammatory bowel disease in the MR analysis using the inverse variance weighted (IVW) method. MR analysis was performed using the R package “TwoSampleMR”. *p*‐values shown are two‐sided and adjusted for multiple comparisons. The gray line indicates that the association remained significant after FDR correction (FDR< 0.05).

Among the 6073 MSDP‐associated CpG sites in newborns, 4217 and 4216 had local methylation quantitative trait loci (*cis*‐mQTLs) that could be used as Instrumental variables (IVs) in the epigenetic MR analysis for CD and UC, respectively. We identified 122 MSDP‐associated CpG sites in newborns that were associated with the risk of CD (FDR corrected *p*< 0·05), such as cg16490823 (*RPS6KA2*, OR = 1·23, 95 %CI: 1·19–1·28), cg02879453 (*ADCY7*, OR = 3·53, 95 %CI: 2·55–4·90), cg14588779 and cg08097581 (*AKAP8L*, OR = 1·09, 95 %CI: 1·04–1·14), cg03644587 (*TNXB*, OR = 2·16, 95 %CI: 1·69–2·76), cg27068095 (*PRRT1*, OR = 1·91, 95 %CI: 1·53–2·37), and cg07404514 (*CLIC1/DDAH2*, OR = 1·70, 95 %CI: 1·41–2·05) (Figure 2 and Table S4). We did not detect any obvious heterogeneity nor horizontal pleiotropy for the instruments used in this analysis through the Q‐test and MR‐Egger intercept (Tables S5 and S6). Regarding the analysis of UC, 115 MSDP‐associated CpG sites in newborns were discovered associating the risk of UC (FDR corrected *p*<0·05), including five CpG sites within *AHRR* (OR = 1·43, 95 %CI: 1·23–1·65), eight CpG sites within *PRRT1* (OR = 3·61, 95 %CI: 2·32–5·61), cg19089201 (*MYO1G*, OR = 1·16, 95 %CI: 1·06–1·27), cg23384708 (*TNF*, OR = 2·68, 95 %CI: 1·64–4·38), cg16391678 (*ITGAL*, OR = 2·15, 95 %CI: 1·58–2·92), and cg02135488 (*RPS6KA2*, OR = 1·34, 95 %CI: 1·12–1·59) (Figure 2; Table S7). Similarly, no heterogeneity and horizontal pleiotropy were detected for these instruments (Tables S8 and S9).

For the analysis of MSDP‐induced DNA methylation alterations in childhood, all five CpG sites had *cis*‐mQTLs that could be used in the epigenetic MR analysis of CD and UC. However, we found that three MSDP‐associated CpG sites in childhood were associated with the risk of UC (FDR corrected *p*<0·05), i.e., cg19089201 (OR = 1·16, 95 %CI: 1·06–1·27), cg22132788 (OR = 1·13, 95 %CI: 1·04–1·24), and cg12803068 (OR = 1·11, 95 %CI: 1·03–1·20). All of them are located within the *MYO1G* region (Table 2).

**TABLE 2:** Validation of DNA methylation sites at multiple early life stages induced by maternal smoking during pregnancy and associated with the risk of inflammatory bowel disease.

Exposure	Period of DNAm	Gene	CpG	Outcome	Method	nsnp	OR	LCI	UCI	FDR	Validation in IBD EWAS/adult	Validation in colocalization	Validation in intestine	Identified in newborn
MSDP	childhood	*MYO1G*	cg19089201	UC	IVW	4	1·16	1·06	1·27	5·32E‐03	Yes	No	Yes	Yes
			cg22132788	UC	IVW	3	1·13	1·04	1·24	6·78E‐03	Yes	No	Yes	Yes
			cg12803068	UC	IVW	4	1·11	1·03	1·20	6·78E‐03	Yes	No	Yes	Yes
MSDP	adult	*RHOA*	cg00174179	CD	Wald ratio	1	0·26	0·20	0·34	4·03E‐21	Yes	Yes	No	No
MSDP	adult	*GFI1*	cg14179389	CD	Wald ratio	1	0·84	0·76	0·93	1·40E‐02	Yes	No	Yes	Yes
				UC	Wald ratio	1	0·85	0·77	0·94	2·57E‐02	Yes	No	Yes	Yes
MSDP	adult	*FOXK1*	cg14485097	CD	Wald ratio	1	1·47	1·17	1·84	1·40E‐02	Yes	No	No	No
			cg25879142	CD	Wald ratio	1	1·34	1·13	1·60	1·40E‐02	Yes	No	No	No
MSDP	adult	*MYO1G*	cg19089201	UC	IVW	4	1·16	1·06	1·27	2·57E‐02	Yes	No	Yes	Yes
			cg04180046	UC	IVW	4	1·14	1·05	1·24	2·57E‐02	Yes	No	Yes	Yes
			cg22132788	UC	IVW	3	1·13	1·04	1·24	3·49E‐02	Yes	No	Yes	Yes
			cg12803068	UC	IVW	4	1·11	1·03	1·20	3·49E‐02	Yes	No	Yes	Yes
MSDP	adult	*AHRR*	cg17924476	UC	Wald ratio	1	1·18	1·06	1·31	3·49E‐02	Yes	No	Yes	Yes

MSDP, maternal smoking during pregnancy. nsnp, number of instruments. OR, odds ratio. LCI, lower 95 % confidence interval. UCI, upper 95 % confidence interval. FDR, false discovery rate. IBD, inflammatory bowel disease. EWAS, epigenome‐wide association study. UC, ulcerative colitis. CD, Crohn's disease. IVW, inverse‐variance weighted.

Regarding the analysis of altered DNA methylation in adolescence and adulthood (16–48 years old) caused by MSDP, 60 of the 69 CpG sites had available instruments for the next analysis. After multiple testing correction, altered DNA methylation at four CpG sites were associated with the risk of CD, including cg00174179 (*RHOA*, OR = 0·26, 95 %CI: 0·20–0·34), cg14179389 (*GFI1*, OR = 0·84, 95 %CI: 0·76–0·93), and two CpG sites (cg14485097 [OR = 1·47, 95 %CI: 1·17–1·84] and cg25879142 [OR = 1·34, 95 %CI: 1·13–1·60]) located within *FOXK1* region (Table 2). Additionally, seven MSDP‐associated CpG sites in adolescence and adulthood were linked to the risk of UC, i.e., cg14179389 (*GFI1*, OR = 0·85, 95 %CI: 0·77–0·94), cg17924476 (*AHRR*, OR = 1·18, 95 %CI: 1·06–1·31), and four CpG sites located within *MYO1G* region (Table 2).

The analysis of altered DNA methylation in newborns caused by paternal and grandmaternal smoking with the risk of IBD showed no statistical significance (data not shown). These two exposures were excluded from subsequent analyses.

### 2.4 External IBD EWAS and Previous Study Further Confirmed the Effect of MSDP‐Associated DNA Methylation on the Development of IBD in Offspring

When compared with controls, a total of 7809 and 1937 differentially methylated CpG sites were identified for individuals with CD and UC, respectively (Tables S10 and S11). Next, we compared the findings from the current study with those from the IBD EWAS and our previous study, which was conducted in adults (Table S12 and S13).

**FIGURE 3:**
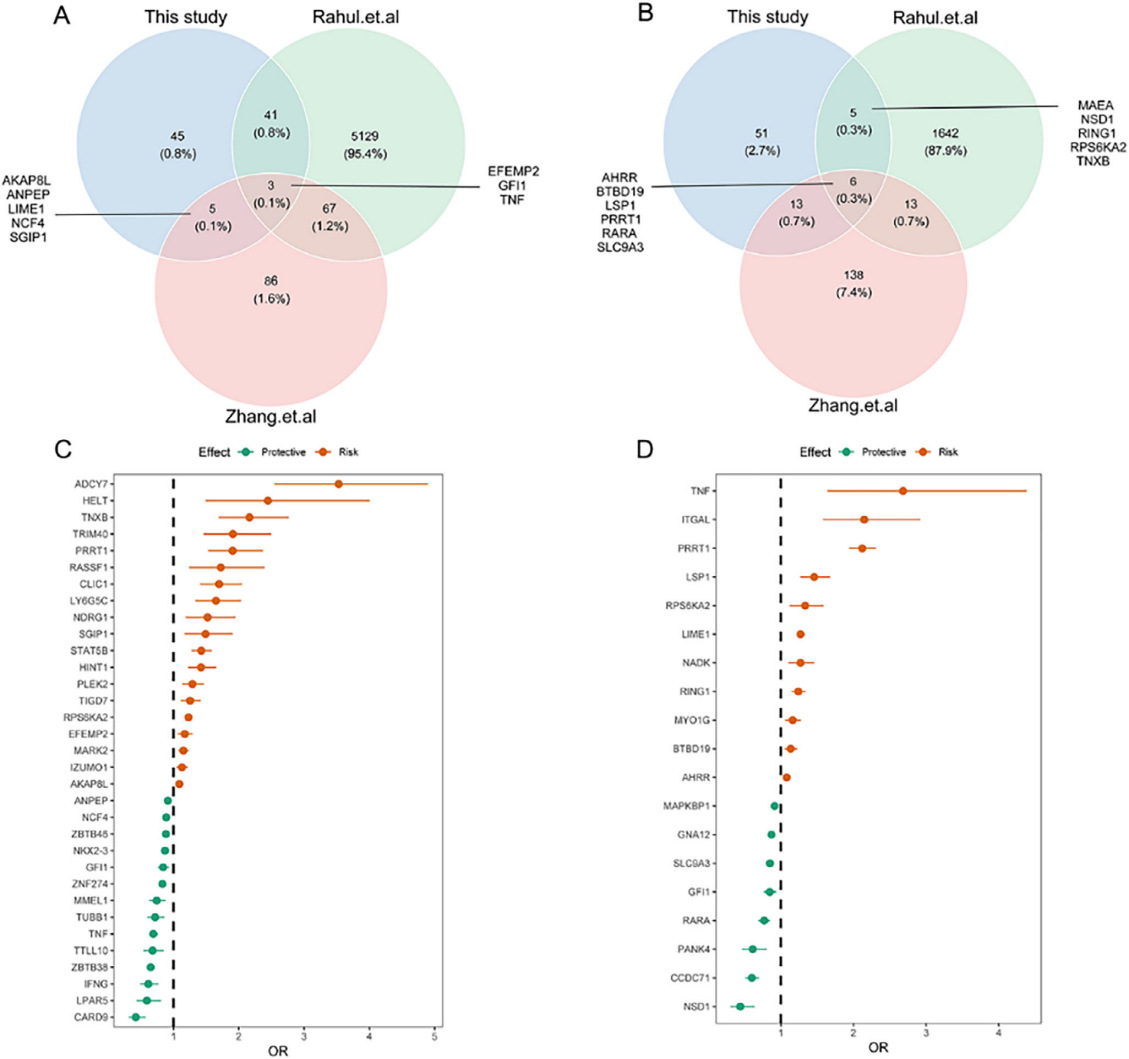
The validation of the significant loci that were identified in Crohn's disease and ulcerative colitis epigenetic Mendelian randomization analysis. A) Venn diagram shows the finding overlap of the current study with external IBD EWAS as well as previous studies (Crohn's disease). B) Venn diagram shows the finding overlap of the current study with external IBD EWAS, as well as the previous study (ulcerative colitis). C) forest plot of significant loci identified in Crohn's disease analysis. D) forest plot of significant loci identified in ulcerative colitis analysis. Data in forest plots are presented as odds ratios (ORs) with 95 % confidence intervals (CIs). All tests were two‐sided, and *p*< 0.05 was considered statistically significant.

For the analysis of MSDP‐associated DNA methylation in newborns, a total of 49 loci were validated for CD in the IBD EWAS or our previous study. Specifically, 44 loci from the current study were validated against the IBD EWAS. Additionally, eight loci were validated against our previous study. Of these, three (*EFEMP2*, *GFI1*, and *TNF*) were common to all three studies (Figure 3A,C). Regarding the replication of UC findings, 24 loci were validated. Particularly, 11 were validated in the IBD EWAS and 19 were replicated in our previous study. Of these, 6 loci were common to all three studies (Figure 3B,D), including *AHRR*, *MYO1G*, *GFI1*, *LSP1*, and *PRRT1*.

Regarding the analysis of MSDP‐associated DNA methylation in childhood (5.5 years old), only *MYO1G*, whose alteration in DNA methylation status is associated with the risk of UC, was replicated in the IBD EWAS and our previous study (Table 2).

For the analysis of MSDP‐associated DNA methylation in adolescents and adults (16–48 years old), three loci (namely *RHOA*, *GFI1*, *FOXK1*) identified in the CD analysis and three loci (namely *GFI1*, *MYO1G*, *AHRR*) discovered in the UC analysis were replicated in this stage (Table 2).

### 2.5 Multiple Validation Analyses Verified the Most Reliable DNA Methylation Sites and Potential Shared Methylation Loci Across Different Life Stages in IBD Pathogenesis

We performed a colocalization analysis for CpG sites replicated in both the external IBD EWAS and our previous study. This was done to strengthen the evidence for the potential link between MSDP‐associated DNA methylation in different offspring life stages and the risk of CD and UC. For MSDP‐associated CpG sites in newborns, five loci (located within *ADCY7*, *IFNG*, *TIGD7*, *AKAP8L*, and *NCF4*) showed colocalization evidence (with a PPH4>0·80) with the genetic susceptibility of CD. Additionally, four loci (located within *AHRR*, *PRRT1*, *GNA12*, *ITGAL*) demonstrated colocalization evidence with UC susceptibility (Figure S9 and Table S14). For those MSDP‐associated CpG sites in childhood (5.5 years old) and adolescent and adulthood (16–48 years old), only one locus (located within *RHOA*, 16–48 years old) showed colocalization evidence with the genetic susceptibility of CD (Table 2; Table S14).

Next, we assessed the regulatory pattern of those CpG sites (validated in the external IBD EWAS and our previous study) on their corresponding genes in the intestinal tissue. For the MSDP‐associated CpG sites in newborns, a total of 11 CpG sites were identified to be associated with the expression of their mapped genes (ten genes) in CD analysis, and 13 CpG sites were related to the expression of their mapped genes (eight genes) in UC analysis (Table 3; Table S15). Concerning the MSDP‐associated CpG sites in adolescents and adults (16–48 years old), three CpG sites were found to be associated with the expression of their mapped genes (namely *GFI1*, *MYO1G*, and *AHRR*) in intestinal tissues. Among these three genes, *GFI1* is a shared locus in both CD and UC

**TABLE 3:** The association between those significant CpG sites and the gene expression level in intestinal tissues.

Exposure	Outcome	Nearest Gene	CpG	effect	se	t	p
MSDP	CD	*CARD9*	cg00853853	−1·038	0·360	−2·884	4·22E‐03
		*CLIC1*	cg07404514	−1·128	0·194	−5·830	1·52E‐08
		*MARK2*	cg21609804	−1·231	0·476	−2·588	1·01E‐02
		*PLEK2*	cg06724236	−1·799	0·768	−2·342	1·99E‐02
		*PRRT1*	cg27068095	1·298	0·371	3·498	5·40E‐04
		*RASSF1*	cg26357744	−11·426	3·746	−3·050	2·49E‐03
		*RPS6KA2*	cg02135488	1·770	0·828	2·137	3·34E‐02
		*RPS6KA2*	cg16490823	1·675	0·429	3·900	1·19E‐04
		*TNXB*	cg03644587	1·567	0·641	2·444	1·51E‐02
		*GFI1*	cg14179389	1·581	0·400	3·951	9·69E‐05
		*TNF*	cg17741993	−1·560	0·659	−2·366	1·86E‐02
MSDP	UC	*AHRR*	cg26954197	0·824	0·366	2·252	2·51E‐02
		*AHRR*	cg26850624	1·864	0·382	4·883	1·70E‐06
		*PRRT1*	cg10551329	−0·580	0·247	−2·349	1·96E‐02
		*RPS6KA2*	cg02135488	1·770	0·828	2·137	3·34E‐02
		*SLC9A3*	cg02354563	−2·240	1·119	−2·002	4·62E‐02
		*SLC9A3*	cg12833872	6·801	1·044	6·511	3·12E‐10
		*SLC9A3*	cg18096398	5·810	1·940	2·995	2·97E‐03
		*SLC9A3*	cg22572362	7·924	0·823	9·634	<2·00E‐16
		*GFI1*	cg14179389	1·581	0·400	3·951	9·69E‐05
		*GNA12*	cg19717773	−1·007	0·225	−4·471	1·10E‐05
		*GNA12*	cg12444411	−0·980	0·222	−4·409	1·45E‐05
		*MYO1G*	cg19089201	6·220	0·468	13·300	<2·00E‐16
		*TNF*	cg23384708	−1·784	0·787	−2·268	2·41E‐02

MSDP, maternal smoking during pregnancy. CD, Crohn's disease. UC, ulcerative colitis.

In addition, a single‐cell RNA sequencing analysis was performed on intestinal tissue to evaluate the expression of those genes that have been replicated in more than two validation stages across diverse intestinal cell types (Figure S10). The single‐cell sequencing results for CD tissues indicated that IFNG was highly expressed in NK/T cells, with its expression in CD NK/T cells being lower than in normal tissues. *NCF4* was primarily expressed in B cells and myeloid cells, with its expression in CD myeloid cells being lower than in normal tissues. Although *AKAP8L* showed high expression abundance in endothelial cells, the difference in expression was not statistically significant. However, in the CD group, *AKAP8L* expression appeared higher in B cells and stromal cells compared to normal, while possibly lower in epithelial cells. *TNF* showed high expression abundance in myeloid cells, with expression in CD myeloid cells being higher than in normal tissues. *GFI1* expression was not very high overall, but it was higher in myeloid cells in normal tissues compared to CD, while in epithelial cells, the expression in CD tissues appeared higher than in normal. (Figures S11–S13). Furthermore, the single‐cell sequencing results for UC tissues indicated that *AHRR* had the highest expression abundance in endothelial cells, but the expression differences were not statistically significant. In addition, *AHRR* expression in myeloid cells, stromal cells, and NK/T cells in the normal group tended to be higher than in the UC tissues. *MYO1G* showed the highest expression in myeloid cells, with higher expression in the UC tissues than in the normal tissues. *GNA12* exhibited relatively high expression in endothelial cells, myeloid cells, and stromal cells, but the differences were not statistically significant. The expression of *GFI1* was not high across various cell subtypes, but the expression in NK/T cells and Plasma cells in the UC tissues was higher than in the normal tissues (Figures S14–S16). Additionally, we assessed whether the key transcriptomic signatures identified in our analysis were consistently observed across individual CD samples rather than being driven by a single patient. The results showed that most of the key genes exhibited largely consistent directions of expression changes across different samples (Figure S17), suggesting that the findings are not solely individual‐specific.

Finally, we compared significant loci identified at diverse life stages with the aim of identifying DNA methylation sites that may be shared by smoking exposure during different life stages in the development of IBD. We found that the alterations of DNA methylation status within *LTA/TNF*, *AHRR*, *MYO1G*, and *GFI1* gene regions induced by smoking exposure at various life stages might represent a shared feature throughout the development of IBD (Table 4; Figure S18).

**TABLE 4:** Shared DNA methylation sites related to smoking exposure at different life stages in inflammatory bowel disease.

Outcome	Gene	MSDP_Newborn		MSDP_Chidhood		MSDP_AA		Adult	
		CpG	OR (95 %CI)	CpG	OR (95 %CI)	CpG	OR (95 %CI)	CpG	OR (95 %CI)
CD	*LTA/TNF*	cg26729380	0·71 (0·64, 0·78)	∖	∖	∖	∖	cg03599224	0·61 (0·50, 0·75)
CD	*LTA/TNF*	cg17741993	0·48 (0·31, 0·75)	∖	∖	∖	∖		
CD	*GFI1*	cg14179389	0·84 (0·76, 0·93)	∖	∖	cg14179389	0·84 (0·76, 0·93)	cg14179389	0·84 (0·76, 0·93)
UC	*AHRR*	cg26954197	1·43 (1·23, 1·65)	∖	∖	cg17924476	1·18 (1·06, 1·31)	cg26954197	1·43 (1·23, 1·65)
UC	*MYO1G*	cg19089201	1·16 (1·06, 1·27)	cg19089201	1·16 (1·06, 1·27)	cg19089201	1·16 (1·06, 1·27)	cg19089201	1·17 (1·08, 1·28)
UC	*GFI1*	cg14179389	0·85 (0·77, 0·94)	∖	∖	cg14179389	0·85 (0·77, 0·94)	cg14179389	0·85 (0·77, 0·94)

CD, Crohn's disease. UC, ulcerative colitis. MSDP, maternal smoking during pregnancy. AA, adolescent and adult.

## 3 Discussion

Early life constitutes a sensitive period for growth and development, exerting a crucial role in the occurrence and progression of numerous diseases in later life, including the susceptibility to developing IBD. As exposure to tobacco smoking in early life is believed to represent a crucial element in shaping the risk of IBD, we assessed this association via a prospective cohort study and updated meta‐analysis. We discovered that exposure to MSDP was potentially correlated with increased risks of IBD, CD, and UC, and that this particularly manifested in adulthood. In addition, active smoking during adolescence was associated with an increased likelihood of developing IBD and CD in later life. Through integrating multi‐omics data, we further identified DNA methylation changes associated with MSDP at several loci that may be involved in the development of CD and UC across different life stages. Specifically, alterations in DNA methylation within *PRRT1*, *ITGAL*, *AHRR*, and *GNA12* were potentially linked to UC risk, and DNA methylation alterations within *PRS6KA2*, *SLC9A3*, *GFI1*, *MYO1G*, and *TNF* might also play a role in the pathogenesis of UC. For CD, several loci with potential involvement were identified, including *TIGD7*, *IFNG*, *AKAP8L*, *ADCY7*, *NCF4*, *PRS6KA2*, *TNF*, *GFI1*, *TNXB*, amongst others. Additionally, we discovered that the alterations of DNA methylation status within the gene regions of *AHRR*, *MYO1G*, *LTA/TNF*, and GFI1 might be the shared pathogenesis through which smoking exposure during different life stages contributes to IBD development.

The association between early life exposure to tobacco smoking and the risk of IBD has been examined in previous studies with inconsistent findings. These studies, however, were limited by sample size and heterogeneity. [8, 9, 14] Furthermore, most previously performed studies have focused on passive smoking during early life, and evidence supporting the association between active smoking before adulthood and IBD risk is limited. In a large‐scale prospective cohort study, we found that personal active smoking during adolescence was positively correlated with the risk of CD, and indicated a potential protective effect of pre‐adulthood active smoking on UC development in those aged over 60 years. This finding is in line with several previous studies [28, 31] despite some differences in study designs. These findings also align with established epidemiological evidence in adult populations, wherein smoking is consistently associated with an elevated risk of CD yet exhibits a paradoxical protective effect against UC. By performing our large sample size prospective cohort study with meta‐analysis, we strengthen the evidence that MSDP is a risk factor for IBD in offspring. In line with our current study, a meta‐analysis conducted in 2021 in 525770 individuals reported a positive association between MSDP and IBD risk. [32] However, subgroup analyses stratified by disease subtypes (CD and UC) revealed no statistically significant associations. [32] The included studies in that meta‐analysis encompassed both case‐control and cohort designs, and most of the studies were conference abstracts. Moreover, the inclusion of both CD and UC under the umbrella term “IBD” in their analysis may have introduced substantial heterogeneity and potential publication bias. This methodological approach carries significant limitations, as the distinct pathophysiological characteristics of CD and UC could lead to disease misclassification, thereby compromising the reliability of the meta‐analytic findings. In contrast, our study included high‐quality cohort studies only, further demonstrating the potential association between maternal smoking during pregnancy and the risks of IBD, CD, and UC. The heterogeneity observed in the IBD analysis was mainly driven by Omer Karur et al. (2021), likely reflecting its distinct exposure assessment methodology compared with other included cohorts. In addition to MSDP, prenatal smoking exposure and passive smoking exposure during childhood have also been linked to the risk of CD and UC. [10, 11, 12] However, these prior investigations were often limited by small sample sizes and yielded inconsistent results. By pooling these studies, we discovered that prenatal exposure to smoking was a risk factor for CD and UC, and passive smoking exposure during childhood was a contributing factor to CD development. In summary, we reported early‐life smoke exposure to be detrimental to the health of the offspring, in turn emphasizing the need for prevention strategies amongst couples and parents, which is necessary to help control rising IBD incidences globally.

In addition to identifying risk factors for disease, it is equally, if not of greater importance, to explore the mechanisms by which risk factors affect the development of disease. Fetal exposure to tobacco smoke can alter fetal immune homeostasis and induce hypoxia, cause vasoconstriction of the placenta, and lead to epigenetic modifications and alterations in the infant microbiome. [33, 34, 35] Although DNA methylation changes have been implicated in IBD [36, 37], it has not been established whether early life exposure to tobacco smoking could influence the IBD risk by inducing DNA methylation alterations. Using a Mendelian randomization study, along with a series of validation strategies, our findings provide important evidence suggesting that maternal smoking during pregnancy may influence the development of IBD through modulation of DNA methylation at specific loci.

The most significant DNA methylation alterations induced by smoking have been identified in the aryl‐hydrocarbon receptor repressor (AHRR) gene region. The *AHRR* gene can also serve as a dominant marker for tobacco smoking. [38, 39] Both the current study and our previous study demonstrated that DNA methylation alterations within the AHRR region induced by smoking or MSDP are associated with the risk of UC, indicating that the DNA methylation changes in this region may be stable across different life stages. Mounting evidence positions *AHRR* as a pivotal regulator of intestinal inflammation. First, AHRR modulates innate immunity (NF‐κB) and antimicrobial protein genes while preventing AHR‐driven immunosuppression and autoimmunity (e.g., IBD). [40, 41] Crucially, *AHRR* maintains intestinal intraepithelial lymphocytes (IELs); its deficiency induces CYP1A1‐mediated oxidative stress, triggering ferroptosis in IELs and exacerbating colitis in *AHRR* ^−/−^ mice. [42] Notably, IBD‐associated *AHRR* DNA methylation changes in intestinal tissue [43] further underscore its pathogenic role, suggesting *AHRR* as both a biomarker and therapeutic target. Given the robust association of *AHRR* with smoking and its potential role in IBD pathogenesis, future research should elucidate the mechanisms through which *AHRR* influences IBD, thereby facilitating the development of targeted prevention and treatment strategies.

In addition to *AHRR*, we also identified DNA methylation changes associated with MSDP within the G protein subunit alpha 12 (GNA12) and proline‐rich transmembrane protein 1 (PRRT1) region as the primary evidence related to the risk of UC and/or CD. *GNA12* encodes guanine nucleotide‐binding protein (G protein) alpha 12, a membrane‐bound GTPase that plays a crucial role in the assembly of tight junctions in epithelial cells by interacting with ZO‐1 and Src. [44] It has been reported to be linked with UC risk through regulating intestinal barrier function. [45, 46] The *PRRT1* gene is situated within the class III region of the major histocompatibility complex (MHC), adjacent to the boundary of the class II region. It is a non‐classical human leukocyte antigen (HLA) gene that frequently serves as a focal point in research on immune‐related disorders. The HLA region is extensively recognized as a regulatory locus for a diverse range of diseases, [47] including IBD. [7, 48] Importantly, the same HLA region has been highlighted in previous studies on smoking, environmental pollution, and independent IBD EWAS datasets. [7,49, 50] This repeated observation across diverse studies underscores its potential role in mediating environmental effects on IBD and warrants further mechanistic investigation. Therefore, elucidating whether it is the *PRRT1* gene or the MHC region that genuinely contributes to the pathogenesis of IBD represents a highly intriguing research avenue. Additionally, *PRRT1* constitutes a component of native AMPA receptor (AMPAR) complexes in multiple brain regions. Its product can retard the inactivation and desensitization of synaptic glutamate receptor AMPA regulation, which is indispensable for synapse development and function. [51] AMPAR is a tetrameric ligand‐gated ion channel that mediates the transmission of glutamate signals in the brain, where glutamate was found to serve not only as a neurotransmitter but also as an immunomodulator. [52, 53] Moreover, it was discovered that the overexpression of *PRRT1* could enhance neuronal viability, decrease apoptosis, and exert an impact on autophagy. [54] Dysregulated autophagy has been implicated in CD pathogenesis. [37] Although the mechanism by which *PRRT1* is implicated in the pathogenesis of IBD remains unclear, immune pathways, autophagy, and the brain‐gut axis may offer a potential explanation. Overall, more studies are needed to further explore the role of *GNA12* and *PRRT1* in UC and/or CD development.

In this study, we also identified other DNA methylation changes caused by MSDP within several gene regions that were associated with the risk of UC and CD, including *TNF*, *GFI1*, and *RPS6KA2*. Notably, *TNF* and *GFI1* are shared loci of different life stage exposure to smoking in the pathogenesis of IBD. *TNF* is also situated in the MHC region and appears to be the principal pro‐inflammatory cytokine implicated in the development of IBD. [2] It promotes inflammation by inducing the production of other pro‐inflammatory cytokines, such as IL‐1β and IL‐6. [2] *TNF* also stimulates the uptake of epithelial antigens in the ileum. [2] TNF is one of the most well‐established IBD risk genes, supported by both GWAS and extensive experimental evidence. [55, 56, 57] Although anti‐TNF‐directed therapy has been extensively utilized for the treatment of IBD in clinical practice, there is currently no single “gold standard” for evaluating the efficacy of anti‐TNF therapy in patients with CD or UC. [58] Therefore, the discovery of reliable biomarkers, such as DNA methylation, could be of paramount significance for clinical practice. Growth Factor Independent 1 Transcriptional Repressor (GFI1) encodes a nuclear zinc finger protein that functions as a transcriptional repressor and is of crucial significance for the development and function of immune and hematopoietic cells. Although no studies have directly linked GFI1 to IBD, its critical role in immune regulation suggests it may influence immune processes relevant to IBD. [57, 58, 59] Functionally, *GFI1* is capable of regulating neutrophil differentiation, facilitating lymphoid cell proliferation, and is indispensable for granulocyte development. [62] It can also inhibit the transcriptional activity of the macrophage‐specific gene *SPI1* by means of similarity, suppress the differentiation of macrophage myeloid progenitor cells, promote granulocyte‐directed differentiation, and mediate the selective splicing of CD45 in conjunction with *U2AF1L4* and control T cell receptor signaling. Additionally, *GFI1* can also counteract *RELA* to regulate endotoxin‐mediated toll‐like receptor (TLR) inflammatory responses. [63] Given these functions, GFI1 represents a biologically plausible contributor to IBD immune dysregulation, despite the lack of direct human genetic evidence. *RPS6KA2* is a member of the RSK (ribosomal S6 kinase) family of serine/threonine kinases. Our group and others have consistently implicated this gene in a series of studies on the IBD methylome. [37, 64, 65, 66] This finding suggests that DNA methylation could be a mechanism through which genetic variants outside of protein‐coding regions contribute to the disease phenotype. A common feature of this family is the presence of kinase catalytic domains in all its members, enabling them to phosphorylate various molecules associated with the mitogen‐activated protein kinase (MAPK) signaling pathway. [67] The MAPK pathway represents one of the central pathways in the inflammatory response and exerts a crucial role in the pathogenesis of IBD. [68] In addition, *RPS6KA* participates in multiple stages of translational control and acts as a mediator in the PI3K/Akt/mTOR pathway. [69] mTOR‐dependent autophagy, together with its regulatory axis TLR4‐MyD88‐MAPK and NF‐κB, could potentially serve as an alternative therapeutic target for patients suffering from gut inflammation. [70] Taken together, it represents a highly promising avenue to elucidate the specific molecular pathways of *GFI1* and *RPS6KA* in the pathogenesis of IBD, which may facilitate the development of relevant biomarkers and targeted therapeutics.

We also observed that MSDP‐induced DNA methylation change within the *MYO1G* region across different life stages is associated with the risk of UC, suggesting *MYO1G* is another potential and stable biomarker for UC prevention and therapy. *MYO1G* is a class I myosin associated with the plasma membrane, which is abundantly present in T and B lymphocytes as well as mast cells. [71, 72] It has been identified as a potential DNA methylation marker for smoking, MSDP, second‐hand smoke during childhood, and prenatal smoke exposure. [73, 74, 75] Although *MYO1G* is not an established IBD susceptibility gene, its consistent epigenetic responsiveness to smoking and its expression in key immune cell types support potential relevance for UC pathogenesis. Lastly, further exploration into the role of *TIGD7*, *AKAP8L*, *ADCY7*, *NCF4*, and *IFNG*, discovered in CD, and *ITGAL* and *SLC9A3* found in UC, is necessary, as some of them have been demonstrated to be associated with CD or UC in previous studies. [46] A subset of MSDP‐associated CpG sites identified in our analysis overlaps with candidate cis‐regulatory elements at *CARD9*, *TNXB*, and *SLC9A3*, as reported by ENCODE. [76]Although functional evidence is currently lacking, these observations suggest that MSDP‐related methylation changes at these loci may influence gene regulatory processes relevant to IBD. Taken together, our findings provide a robust set of candidate loci for future functional studies.

## 4 Strength and Limitation

The most prominent strength of the current study resides in the integration of observational study, meta‐analysis, epigenetic MR analysis, and a series of multi‐omics evidence to define the potential role of DNA methylation in the association between early life smoking exposure and the risk of IBD, thereby enhancing the reliability of the findings. Second, we investigated whether there are smoking‐related DNA methylation sites that are common across different life stages in the pathogenesis of IBD. This exploration may offer a novel insight for comprehending the potential shared mechanisms through which risk factors at various life stages influence the susceptibility to IBD and builds on our recent work. [7, 48] In addition, it may also present the possibility of identifying potential diagnostic and therapeutic targets for IBD at the earliest stage of exposure to risk factors. In addition to the strengths, there are also some limitations that should be acknowledged. First, since MSDP data were obtained from self‐reported questionnaires completed by UK Biobank participants, potential recall bias cannot be ruled out. This could potentially result in the misclassification of MSDP, which may, in turn, influence the evaluation of its potential impacts on offspring IBD. To address this concern and maximize the accuracy of the assessment, we subsequently carried out a more comprehensive meta‐analysis. Moreover, given that the subsequent EWAS and epigenetic MR analyses were not executed using the UK Biobank dataset, it is improbable that the recall bias inherent in the UK Biobank study would significantly affect the downstream analyses. Second, given the age of the participants in the UK Biobank, we were unable to assess the impact of early‐life smoking on the risk of IBD before adulthood. Nevertheless, we conducted an age‐stratified analysis, and the results indicated a more significant effect of personal smoking prior to adulthood on the risk of CD. Third, the assessment of the effect of various types of early life smoking exposure on the risk of IBD in the primary meta‐analysis was limited by the small number of available cohort studies. Although Egger's test suggested potential publication bias for UC, its reliability is constrained by the limited number of included studies. To address these limitations and further explore the robustness of the findings, we subsequently conducted a secondary meta‐analysis that incorporated both cohort and case‐control studies, thereby expanding the evidence base. Moreover, in the absence of available GWAS data of pediatric IBD and active smoking during adolescence, we were unable to evaluate the influence of MSDP‐induced DNA methylation alterations on pediatric IBD and the role of DNA methylation in the association between active smoking during adolescence and IBD. This limitation highlights critical needs for future longitudinal birth cohort studies with serial epigenetic profiling. Fourth, a clear limitation of this study is the absence of matched genotype data for the scRNA‐seq samples, which prevents direct assessment of whether these individuals carry MSDP‐associated cis‐mQTLs/SNPs. Fifthly, another limitation of our study is the cross‐tissue inference, as the methylation signals were derived from blood, whereas the single‐cell analyses were conducted in intestinal tissues, making it impossible to fully account for the tissue‐ and cell‐type specificity of epigenetic regulation. Finally, beyond statistical and analytical aspects, biological factors must also be considered when interpreting these data. Methylation changes are cell‐type specific, and immune cell subtypes can potentially be modified over time as a result of smoking, disease activity, or drug therapy. Such alterations may influence the observed smoking‐related changes in DNA methylation and transcription. [77] Given that the smoking‐related DNA methylation in CpG sites was derived from cross‐sectional EWASs, we were unable to assess the temporal course, kinetics, and dynamics of smoking on DNA methylation. Further longitudinal investigations to explore the potential impact of smoking on DNA methylation over time will be required. Additionally, some of the differences observed in the single‐cell analyses were modest and accompanied by statistical uncertainty, indicating that these findings should be considered exploratory rather than conclusive.

## 5 Conclusion

In summary, our study demonstrates that MSDP may constitute a risk factor for IBD in the offspring, and it may exert its effect by modifying the DNA methylation status within certain loci. Moreover, active smoking during adolescence may increase the risk of CD and IBD in later life. Our study also points toward shared DNA methylation pathways across different life stages through which smoking exposure exerts a stable influence on the risk of IBD. These findings carry significant implications for the prediction of disease development, targeted prevention, and novel targets for treatment strategies.

## 6 Methods

### 6.1 Study Design

**FIGURE 4:**
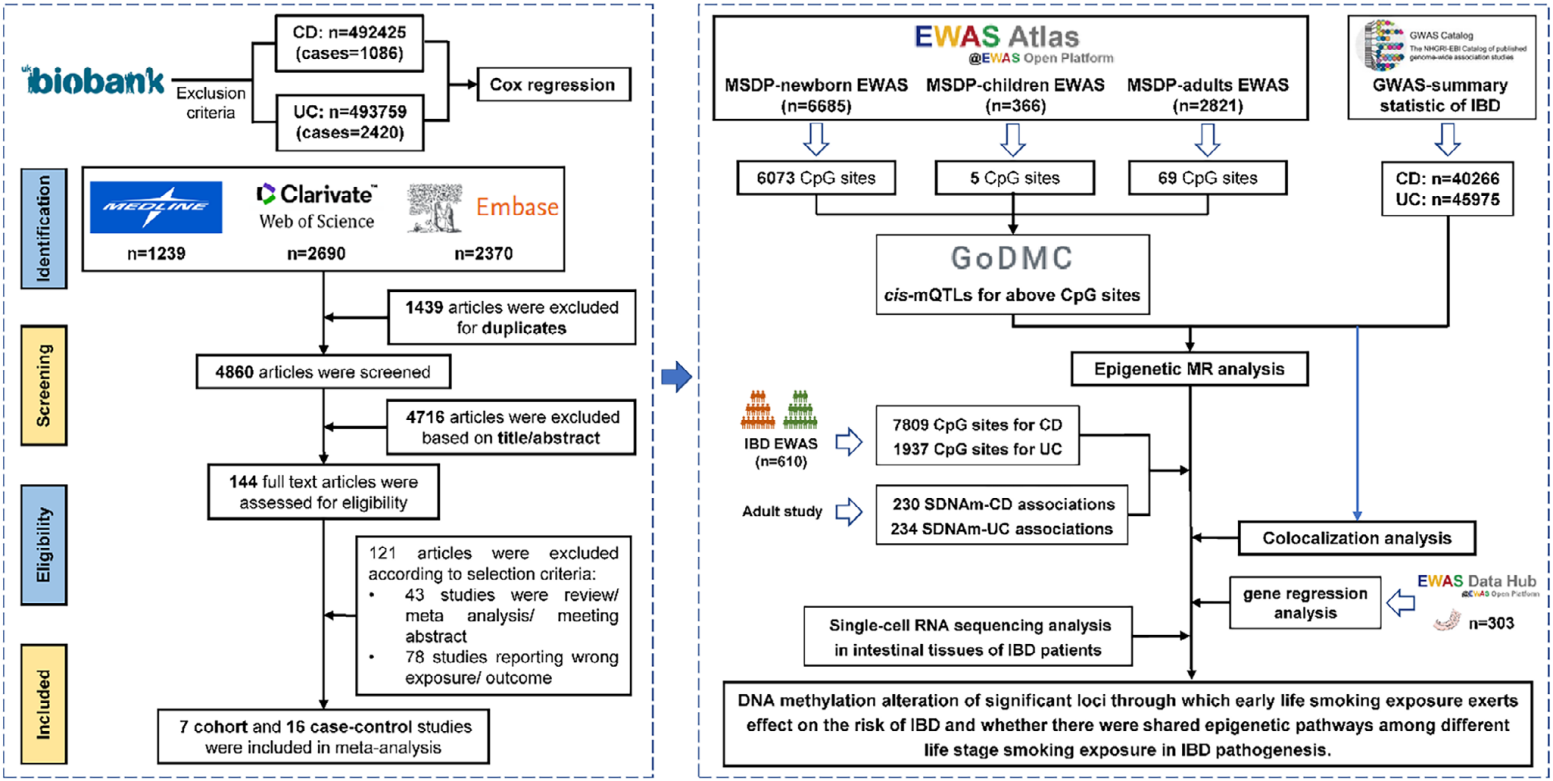
The flow chart of this study. MSDP, maternal smoking during pregnancy. EWAS, epigenome‐wide association study. GWAS, genome‐wide association study. CD, Crohn's disease. UC, ulcerative colitis. MR, Mendelian randomization. The logos of the UK Biobank (https://www.ukbiobank.ac.uk), Web of Science (https://www.webofscience.com), Embase (https://www.embase.com), EWAS Atlas (https://ngdc.cncb.ac.cn/ewas/atlas), GWAS Catalog (https://www.ebi.ac.uk/gwas/home), and GoDMC (http://mqtldb.godmc.org.uk) were retrieved by capturing screenshots from their respective public homepages.

In this study, we first conducted a cohort study based on the UK Biobank to evaluate the impact of MSDP and active smoking during childhood as well as adolescence on the risk of IBD. Next, we evaluated the association between early life exposure to tobacco smoking and the risk of IBD through an updated meta‐analysis. Second, we explored the potential role of DNA methylation associated with early life smoking exposure in the pathogenesis of IBD by carrying out an epigenetic MR analysis. Significant loci identified in the epigenetic MR analysis were validated by an external epigenome‐wide association study of IBD, colocalization analysis, as well as a gene expression analysis and single‐cell analysis in the intestinal tissues. Finally, we performed a comparative analysis of the findings from this study and our previous research in adults to investigate potential shared epigenetic pathways between early life and adult exposure to smoking in the pathogenesis of IBD. The study design is shown in Figure 4.

### 6.2 Prospective Cohort Study in the UK Biobank

#### 6.2.1 Ascertainment of Exposure, Outcome, and Covariates

In this study, whether participants were exposed to MSDP and the age of personal smoking initiation were the primary exposures, with the incidence of CD, UC, and IBD being the disease outcomes. Recruitment age, gender, Townsend deprivation index (TDI), assessment center, aspirin use at baseline, diabetes mellitus and arterial hypertension at baseline, drinking status, active smoking status (only for MSDP), physical activity, diet score, body mass index (BMI), and the first ten genetic principal components were employed as covariates. [9] Participants with available information on main exposures and covariates were included in the exposure group, while subjects who were never exposed to MSDP or never smoked were regarded as the control group. The incidence cases of IBD, CD, and UC were defined by the 9th and 10th international classification of disease codes. The details of participants' selection are described in the Supporting Information.

#### 6.2.2 Statistical Analysis

A total of 495,075 (3736 incident cases), 492,425 (1086 incident cases), and 493,759 (2420 incident cases) individuals were finally included in the analyses for IBD, CD, and UC, respectively. The age of smoking initiation was categorized into childhood (5–12 years old), adolescence (13–18 years old), and adulthood (>18 years old). When characterizing the demographic characteristics of the participants, continuous variables with a normal distribution were presented as mean ± standard deviation, while categorical variables were expressed as percentages. Cox regression models were employed to evaluate the association between the age of personal smoking initiation and the risk of CD and UC. Model 1 was adjusted for recruitment age, sex, assessment center, BMI, TDI, aspirin use at baseline, and diabetes mellitus and arterial hypertension at baseline, while model 2 was further adjusted for the other covariates mentioned above. A subgroup analysis was conducted in two age groups (≤ 60 and >60 years old) to explore the effect of the age of personal smoking initiation on early‐ and late‐onset IBD. All analyses were carried out using R software (version 4.2.2), and all tests were two‐sided. A *p*‐value< 0.05 was considered statistically significant.

### 6.3 Meta‐Analysis of Cohort Studies

#### 6.3.1 Study Identification

The protocol for the current meta‐analysis was registered in PROSPERO (register ID: CRD42024595826) and reported according to the Preferred Reporting Items for Systematic Reviews and Meta‐analysis checklist. [78] Early life in this study was defined as pre‐adult, encompassing preconception, pregnancy, infancy, childhood, and adolescence. Two investigators (HZ and JZ) searched three electronic databases, including Medline, Embase, and Web of Science, from inception to November 2024, independently to identify the cohort and case‐control studies that investigated the association of early life exposure to tobacco smoking with the risk of IBD. We adopted a 3‐step parallel review of title, abstract, and full text to identify eligible studies. The current cohort study in the UK Biobank was also included in the meta‐analysis. From each eligible study, two reviewers (HZ and JC) extracted the essential information for the meta‐analysis and evaluated their quality according to the Newcastle–Ottawa Scale (NOS, http://www.ohri.ca/programs/clinical_epidemiology/oxford.asp). The characteristics and quality assessment of all included studies were presented in Tables S1 and S16.

#### 6.3.2 Statistical Analysis

A fixed‐effects or random‐effects meta‐analysis was employed to determine the pooled estimates along with 95 % confidence interval (CI) for eligible cohort studies. Heterogeneity was assessed using the I^2^ score, and we evaluated publication bias through the funnel plot and Egger‐test. Moreover, to enhance the robustness of the primary meta‐analysis, we included all eligible cohort and case‐control studies in the secondary meta‐analysis. All tests were two‐tailed, and *p* < 0.05 was considered statistically significant. The “metafor” package (version 4.6.0) within the R software environment (version 4.2.2) was utilized for performing the meta‐analysis. Supporting Information presents the details of this meta‐analysis.

### 6.4 Epigenetic Mendelian Randomization Analysis

#### 6.4.1 Instrumental Variables Selection for Early Life Tobacco Smoking Exposure

When performing Mendelian randomization (MR) analysis, three key assumptions need to be taken into account: (1) Instrumental variables exhibit a strong correlation with the exposures of interest; (2) The instruments have no association with potential confounders; (3) The selected genetic variants should influence the outcome exclusively through the exposure, without alternative pathways [79]. To identify the CpG sites associated with early life smoking exposure, we conducted a comprehensive screening of the related epigenome‐wide association studies (EWASs) in PubMed and extracted CpGs significantly associated with MSDP, prenatal exposure due to paternal smoking, and prenatal exposure due to grandmaternal smoking from the corresponding EWASs [33, 80, 81, 82, 83]. The details of these EWASs are provided in Table S17. There was no EWAS regarding active or passive smoking exposure prior to adulthood. Notably, previous EWASs have explored the differentially methylated CpG sites of MSDP at different life stages of their offspring. Therefore, we extracted MSDP‐associated CpG sites in accordance with different life stages. Specifically, we identified 6073 CpG sites associated with MSDP in newborns, 5 in 5.5‐year‐old children (childhood), 69 in adolescence and middle‐aged adults (16–48 years old), 21 CpG sites in newborns for prenatal exposure to paternal smoking, and 23 CpG sites in newborns related to prenatal exposure to grandmaternal smoking (Table S18–S22). Next, we retrieved the methylation quantitative trait loci (mQTLs) most strongly associated with each of the above‐mentioned CpG sites from the Genetics of DNA Methylation Consortium (GoDMC) to serve as their instrumental variables (IVs). [84] The details of the GoDMC have been described elsewhere. [84] We selected *cis*‐mQTLs (the distance between mQTL and CpG site<1 MB) which were associated with each CpG site at a genome‐wide significance (*p*< 5 × 10^−8^), and extracted the location, beta, se, and *p*‐value of each cis‐mQTL. Finally, we conducted linkage disequilibrium (LD) pruning (clumping window = 10 000 kb, r^2^ = 0.01) to filter independent instrumental variables for early life exposure to smoking‐associated DNA methylation, and calculated the F‐statistic for each IV to eliminate the weak instruments (F_‐statistic_<10). All selected cis‐mQTLs exceeded this threshold, indicating sufficient instrument strength (Table S23). To ensure consistent gene‐level interpretation, CpG sites were annotated using the standard Illumina Infinium array manifest (450K/EPIC), which provides probe‐specific genomic context based on their physical location in the reference genome. Each CpG was assigned to gene features, including TSS200, TSS1500, 5′UTR, first exon, gene body, or 3′UTR, where applicable. CpGs located outside annotated gene regions were classified as intergenic and mapped to the nearest gene according to genomic distance (nearest‐gene approach). This proximity‐based annotation strategy is the conventional and widely adopted method in EWAS and therefore served as the primary mapping framework in the present study.

#### 6.4.2 GWAS Summary‐Level Data of Inflammatory Bowel Disease

The summary‐level GWAS data for CD and UC were obtained from a large‐scale genome‐wide association study focused primarily on the European population. [82] This study investigated genetic associations in 23,305 subjects and conducted meta‐analysis with previously published summary statistics, resulting in a combined sample size of 59,957 individuals. Among these participants, 12,194 were diagnosed with CD, and 12,366 were diagnosed with UC [85].

#### 6.4.3 Statistical Analysis

In order to investigate the potential causal role of DNA methylation associated with early life smoking exposure in the development of IBD, we considered each CpG site as an exposure and utilized its proxy mQTLs as instrumental variables. Subsequently, we computed the effect estimates of DNA methylation at each CpG site on a per standard deviation (SD) increase in CD or UC risk using the Wald Ratio method. When the number of independent *cis*‐mQTLs for any single CpG site exceeded two, we aggregated the estimates using the inverse‐variance weighted (IVW) approach. Additionally, we employed MR‐Egger and weighted median methods as sensitivity analyses where applicable. Given the large number of CpGs tested, the false discovery rate (FDR) was applied to control for multiple comparisons, and CpGs with FDR < 0.05 were considered statistically significant. The “TwoSampleMR” (version 0.6.8) package within the R software environment (version 4.2.2) was used to perform MR analysis.

### 6.5 Epigenome‐Wide Association Study of Inflammatory Bowel Disease

We performed an EWAS on individuals with CD or UC to identify differentially methylated loci. This EWAS was conducted using the IBD‐CHARACTER inception cohort (EU reference no 305676), which included 154 CD cases, 161 UC cases, and 295 healthy controls. [64] The details of this EWAS study have been described elsewhere. [64] Subsequently, we conducted a comparison of the results obtained from epigenetic MR analysis with those from the IBD EWAS in order to validate the robustness of the identified loci. Given that our specific hypothesis was based on the findings of epigenetic MR, we established a statistical significance threshold of *p*< 0.05 for this stage. In addition, we also compared the findings from the epigenetic MR analysis with our previous study that explored the role of smoking‐related DNA methylation in the association between tobacco smoking and the risk of IBD. [7] The results of the previous study were presented in Table S12 and S13. As noted previously, we also adopted the statistical significance threshold of *p*<0.05 in this process. For multiple CpG sites that were validated in this step and located within the same gene region, we employed an inverse variance‐weighted fixed‐effects model to assess the combined effect of DNA methylation in this genomic region on the risk of CD or UC.

### 6.6 Colocalization Analysis and Gene Expression Analysis in Intestinal Tissue

To investigate whether the association between the DNA methylation level and the risk of IBD was driven by a shared causal variant, a colocalization analysis was carried out for all loci that were validated in the IBD EWAS or in our previous study. To achieve this, all available mQTLs for each CpG site were extracted from the ARIES database and subsequently integrated with the GWAS summary statistics of UC or CD. A summary posterior probability for the CpG site and a posterior probability for the single mQTL utilized as a genetic IV at 80 % or above were considered as evidence of colocalization. The “coloc” R package in R software (version 4.2.2) was utilized to conduct this analysis [86].

We further conducted linear regression analysis to investigate the regulatory pattern of DNA methylation on gene expression in colonic tissue, utilizing data from the EWAS Data Hub (https://ngdc.cncb.ac.cn/ewas/datahub/index). The EWAS Data Hub serves as a valuable resource for aggregating and standardizing DNA methylation array data, encompassing 75,344 samples thus far. It also offers reference DNA methylation profiles across various contexts, including 81 tissues or cell types, 6 ancestry categories, and 67 diseases. [87, 88].

### 6.7 Single‐Cell RNA Sequencing Analysis in Intestinal Tissues

To elucidate the distribution patterns of target genes across distinct cell subtypes, we analyzed their expression profiles in the RNA‐seq dataset GSE214695 using the Seurat package. This dataset comprises colon biopsies obtained from inflamed regions of six UC and six CD patients during routine endoscopic procedures. Additionally, six healthy individuals undergoing colorectal cancer screening, with no evidence of dysplasia or polyps at the time of endoscopy, served as controls. Quality control filtering of cells was performed based on the following criteria: nFeature_RNA between 300 and 70 000, nCount_RNA < 100 000, mitochondrial gene content < 20 %, and red blood cell gene content < 3 %. Doublet cells were identified and removed using the DoubletFinder package. To mitigate batch effects, we applied the ‘IntegrateLayers’ function with the Harmony algorithm following normalization, UMI count scaling, and identification of highly variable features. Subsequently, dimensionality reduction was performed using the Uniform Manifold Approximation and Projection (UMAP) algorithm at a resolution of 0.5. Cell type annotation was conducted using the SingleR package based on well‐established marker genes: endothelial cells, fibroblasts, myeloid cells, B cells, natural killer (NK) cells, T cells, and plasma cells. We then compared target gene expression levels and their proportional distributions across different cell subtypes in UC and CD tissues relative to normal colon tissues. Furthermore, we visualized the gene expression profiles of single‐cell datasets from six CD patients in comparison with healthy controls (Figure S18). Statistical significance was assessed using the Wilcoxon test.

## Author Contributions

X.L., J.S., and E.T. designed and supervised the study. H.Z., J.Z., and J.C. participated in the data curation. H.Z., J.Z., and J.C. performed the data analyses and prepared the tables and figures. X.M., S.Z., A.N., R.K., J.W., C.A., and K.L. contributed to the analysis strategy and data. H.Z. and J.Z. wrote the original draft. K.L. and X.L contributed to project administration. All authors critically revised the content and contributed to editing the paper. X.L is the study guarantor. The corresponding author (X.L) attests that all listed authors meet authorship criteria and that no others meeting the criteria have been omitted.

## Funding

This study is supported by funding that is displayed as follows. Xue Li is supported by the National Nature Science Foundation of China (82470543, 82204019).

## Ethics Statement

The UK Biobank obtained ethical approval from the North West‐Haydock Research Ethics Committee (REC reference: 16/NW/0274). The genome‐wide methylation association study, conducted on a subset of the IBD‐CHARACTER inception cohort, was approved by the Regional Committee for Medical Research Ethics, South‐Eastern Norway (REK sør‐øst 2009/2015) and received endorsement from the privacy protection representative at Akershus University Hospital (13‐123). The ethical approval of published GWASs and EWASs was obtained from their corresponding review board.

## Conflicts of Interest

The authors declare no conflicts of interest.

## Supporting information




**Supporting File 1**: advs73739‐sup‐0001‐SuppMat.docx.

**Supporting File 2**: advs73739‐sup‐0001‐SuppMat.xlsx.

## Data Availability

The data that support the findings of this study are available from the corresponding author upon reasonable request.
