# Supplementary material for: Tobacco Smoke Exposure From Prenatal To Adolescent Periods Drives IBD Pathogenesis: Dynamic DNA Methylation Signatures Across Lifespan Stages
Source: Adv Sci (Weinh). 2026 Jan 11;13(17):e16704. doi: 10.1002/advs.202516704 (PMC13042855; doi:10.1002/advs.202516704)
Supplement: Supplementary file 1 — Supporting File 1: advs73739‐sup‐0001‐SuppMat.docx. [file ADVS-13-e16704-s002.docx]

**Tobacco Smoke Exposure from Prenatal to Adolescent Periods Drives IBD Pathogenesis: Dynamic DNA Methylation Signatures Across Lifespan Stages**

Han Zhang, Jianhui Zhao, Jie Chen, Xinyi Ma, Siyun Zhou, Alexandra Noble, Rahul Kalla, Judith Wellens, Kun Liu, Evropi Theodoratou, * Jack Satsangi, * and Xue Li *

**Content of Online-Only Material**

**eMethods**

**eFigure 1**. The funnel plot and sensitivity analysis of maternal smoking during pregnancy with the risk of inflammatory bowel disease in offspring.

**eFigure 2**. The results of meta-analysis of maternal smoking with the risk of Crohn’s disease in offspring.

**eFigure 3.** The results of meta-analysis of maternal smoking with the risk of overall inflammatory bowel disease in offspring.

**eFigure 4.** The results of meta-analysis of maternal smoking with the risk of ulcerative colitis in offspring.

**eFigure 5**. The results of meta-analysis of passive smoking exposure during childhood with the risk of Crohn’s disease.

**eFigure 6.** The results of meta-analysis of passive smoking exposure during childhood with the risk of ulcerative colitis.

**eFigure 7**. The results of meta-analysis of prenatal smoking exposure with the risk of Crohn’s disease in offspring.

**eFigure 8.** The results of meta-analysis of prenatal smoking exposure with the risk of ulcerative colitis in offspring.

**eFigure 9.** The region colocalization plot of those significant loci which identified in the epigenetic Mendelian randomization analysis.

**eFigure 10.** The annotation of cell types in intestinal of individuals with inflammatory bowel disease.

**eFigure 11**. The global expression of significant loci in intestinal tissues of individuals with Crohn’s disease.

**eFigure 12**. The expression of significant loci in specific intestinal cells of individuals with Crohn’s disease.

**eFigure 13.** The percentage and expression differences of significant loci in specific cells of individuals with Crohn’s disease.

**eFigure 14.** The global expression of significant loci in intestinal tissues of individuals with ulcerative colitis.

**eFigure 15.** The expression of significant loci in specific intestinal cells of individuals with ulcerative colitis.

**eFigure 16.** The percentage and expression differences of significant loci in specific cells of individuals with ulcerative colitis.

**eFigure 17.** Gene expression profiles of different cell subpopulations from six CD patients compared with healthy controls.

**eFigure 18.** Shared DNA methylation sites related to smoking exposure at different life stages in inflammatory bowel disease.

**eMethods**

**The population-based prospective cohort study**

*Study population*

The UK Biobank comprises approximately 500,000 individuals aged between 40 and 69 years who were recruited from a total of 22 assessment centers located across the United Kingdom during the period spanning from 2006 to 2010. It provides a wide range of health-related phenotypic and genotypic information for each individual, including demographic data, lifestyle factors, physical examinations, biological samples, and genetic profiling. The UK Biobank obtained ethical approval from the North West-Haydock Research Ethics Committee (REC reference: 16/NW/0274).

*Maternal smoking during pregnancy, personal smoking initiation age and covariates*

Maternal smoking during pregnancy (MSDP) was reckoned as a binary variable. The age of smoking initiation was included and categorized into childhood (5-12 years old), adolescence (13-18 years old), and adulthood (>18 years old) in the observational study. Additionally, covariates such as recruitment age, gender, town deprivation index (TDI), assessment center, aspirin use at baseline, diabetes and hypertension at baseline, drinking status, personal smoking status (only for MSDP), physical activity, diet score, body mass index (BMI), and the first ten genetic principal components were considered. The information on these factors was acquired from questionnaires at baseline and follow-up, physical measurements, and health registries.

*Ascertainment of inflammatory bowel disease*

Incident IBD, CD, and UC cases with comprehensive data on MSDP, smoking initiation age, and covariates were identified through electronic record of the UK Biobank. Complete follow-up was tracked to April 1, 2024. CD and UC cases were ascertained by using the 9^th^ (555, 5550, 5551, 5552, 5559 for CD; 556, 5569 for UC) and 10^th^ (K500, K501, K508, K509 for CD; K510, K512, K513, K514, K515, K518, K519 for UC) International Classification of Disease codes. Briefly, Participants without complete information on smoking behaviors, covariates, and genetic information, or IBD diagnosis within the first 2 years of follow-up were excluded from the dataset. With a median follow-up of 13.9 years, a total of 495,075 (3736 incident cases), 492,425 (1086 incident cases), and 493,759 (2420 incident cases) individuals were finally included in the analyses for IBD, CD, and UC, respectively.

**Meta-analysis**

*Literature search*

The protocol for the current meta-analysis was registered in PROSPERO (register ID: CRD42024595826) and reported according to the Preferred Reporting Items for Systematic Reviews and Meta-analysis checklist. Two investigators (HZ and XM) searched three electronic databases, including Medline, Embase, and Web of Science, from inception to 1^st^ November 2024 independently to identify the cohort and case-control studies which investigated the association of early life exposure to tobacco smoking with the risk of inflammatory bowel disease. The key items used for literature search was as follows: “((((((((early life) OR (maternal)) OR (pregnancy)) OR (grandmaternal)) OR (father)) OR (child)) OR (adolescent)) or (prenatal)) AND (((smoking) OR (smoke)) OR (tobacco)) OR (cigarette))) AND ((((((inflammatory bowel disease) OR (IBD)) OR (ulcerative colitis)) OR (UC)) OR (Crohn's disease)) OR (CD))”. We adopted a 3-step parallel review of title, abstract, and full text to identify eligible studies, and we also screened the relevant systematic review and meta-analysis papers to identify additional eligible studies. Any discrepancies were resolved by discussion or the third investigator (XL).

*Inclusion and exclusion criteria*

Studies meeting the specified criteria are eligible for inclusion in this study, with early life defined as pre-adolescence encompassing preconception, pregnancy, infancy, childhood and adolescence. Eligible studies must fulfill the following criteria: 1) investigation of the association between early life exposure to tobacco smoking and the risk of IBD; 2) reporting of appropriate estimates such as odds ratio (OR), hazard ratio (HR), relative risk (RR), and 95% confidence interval (95%CI), or provision of data enabling calculation of these estimates; 3) utilization of a cohort or case-control study design. Meanwhile, these studies will be excluded if they: 1) are in the form of meeting abstracts, reviews, meta-analyses, or letters; 2) did not report or provide the required estimates; 3) the full-text is not available. To enhance the robustness of this meta-analysis, only cohort studies were incorporated in the primary meta-analysis, while cohort and case-control studies were simultaneously included in the secondary analysis.

*Data extraction and quality assessment*

From each eligible study, two reviewers (HZ and XM) extracted the information on first author’s name, year, region, exposure, the period of exposure, outcome, the period of outcome, study design, number of cases and total participants, and risk estimates along with 95% confidence interval (CI). In addition, we evaluated the quality of each study by using the Newcastle-Ottawa Scale (NOS, <http://www.ohri.ca/programs/clinical_epidemiology/oxford.asp>).

*Statistical analysis*

A fixed-effects or random-effects meta-analysis was conducted to determine the pooled estimates along with 95% confidence interval (CI) for eligible cohort studies. In a secondary analysis, all eligible studies (cohort and case-control studies) were included and the pooled estimates were calculated based on either fixed-effects or random-effects meta-analysis. Heterogeneity was assessed using the I^2^ score, where an I^2^>50% indicates significant heterogeneity. Additionally, we evaluated publication bias through funnel plot and Egger-test. All tests were two-tailed and *p* < 0.05 was considered statistically significant. The "metafor" package (version 4.6.0) within the R software environment (version 4.2.2) was utilized for performing the meta-analysis.


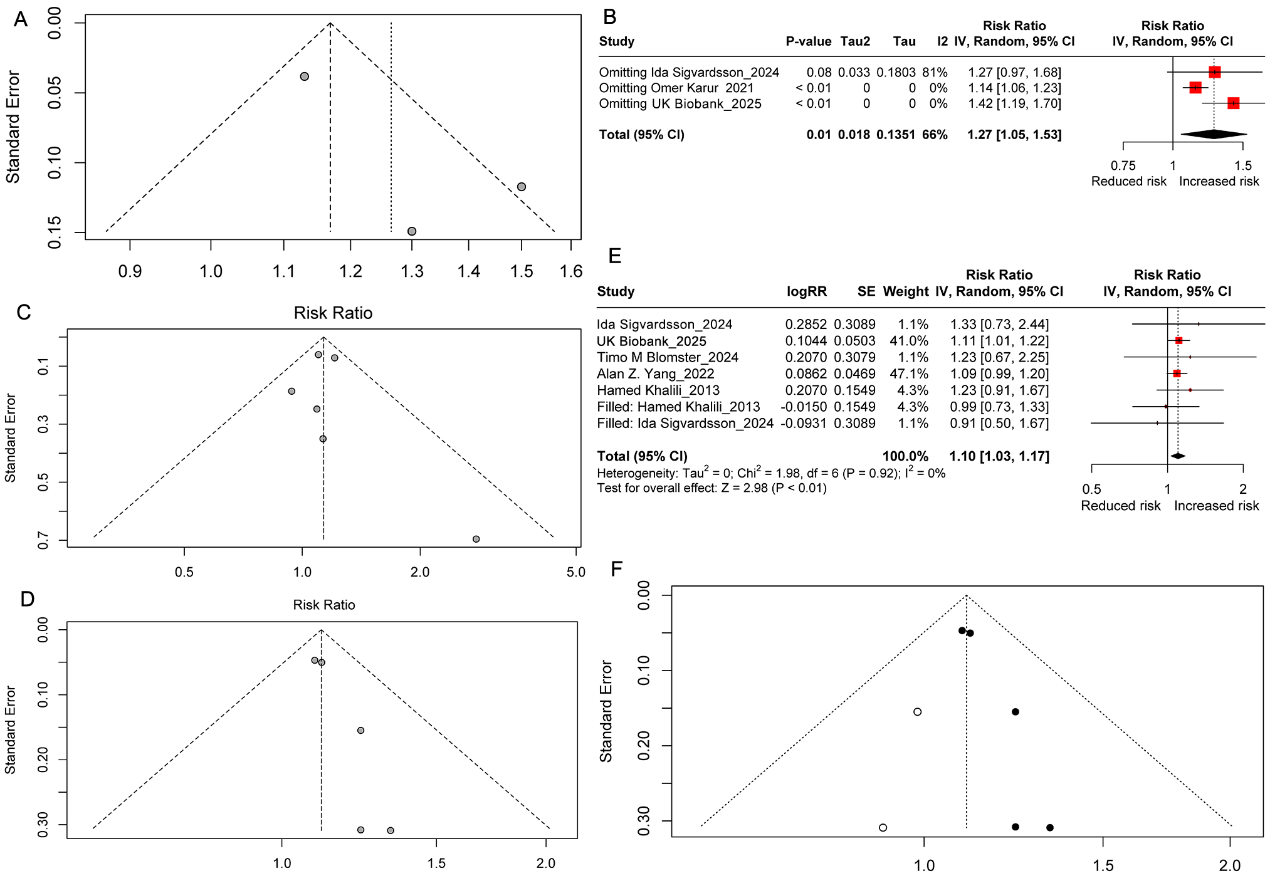


**eFigure 1. The funnel plot and sensitivity analysis of maternal smoking during pregnancy with the risk of inflammatory bowel disease in offspring.** A, funnel plot of maternal smoking during pregnancy and IBD risk. B, leave-one-out analysis of maternal smoking during pregnancy and IBD risk. C, funnel plot of maternal smoking during pregnancy and CD risk. D, funnel plot of maternal smoking during pregnancy and UC risk. E, Trim-and-Fill method results of maternal smoking during pregnancy and UC risk. F, funnel plot of Trim-and-Fill method of maternal smoking during pregnancy and UC risk. Data are presented as odds ratios (ORs) with 95% confidence intervals (CIs) using the inverse-variance (IV), random-effects method. Meta-analyses were performed using the R package “metafor.” All tests were two-sided, and p < 0.05 was considered statistically significant.


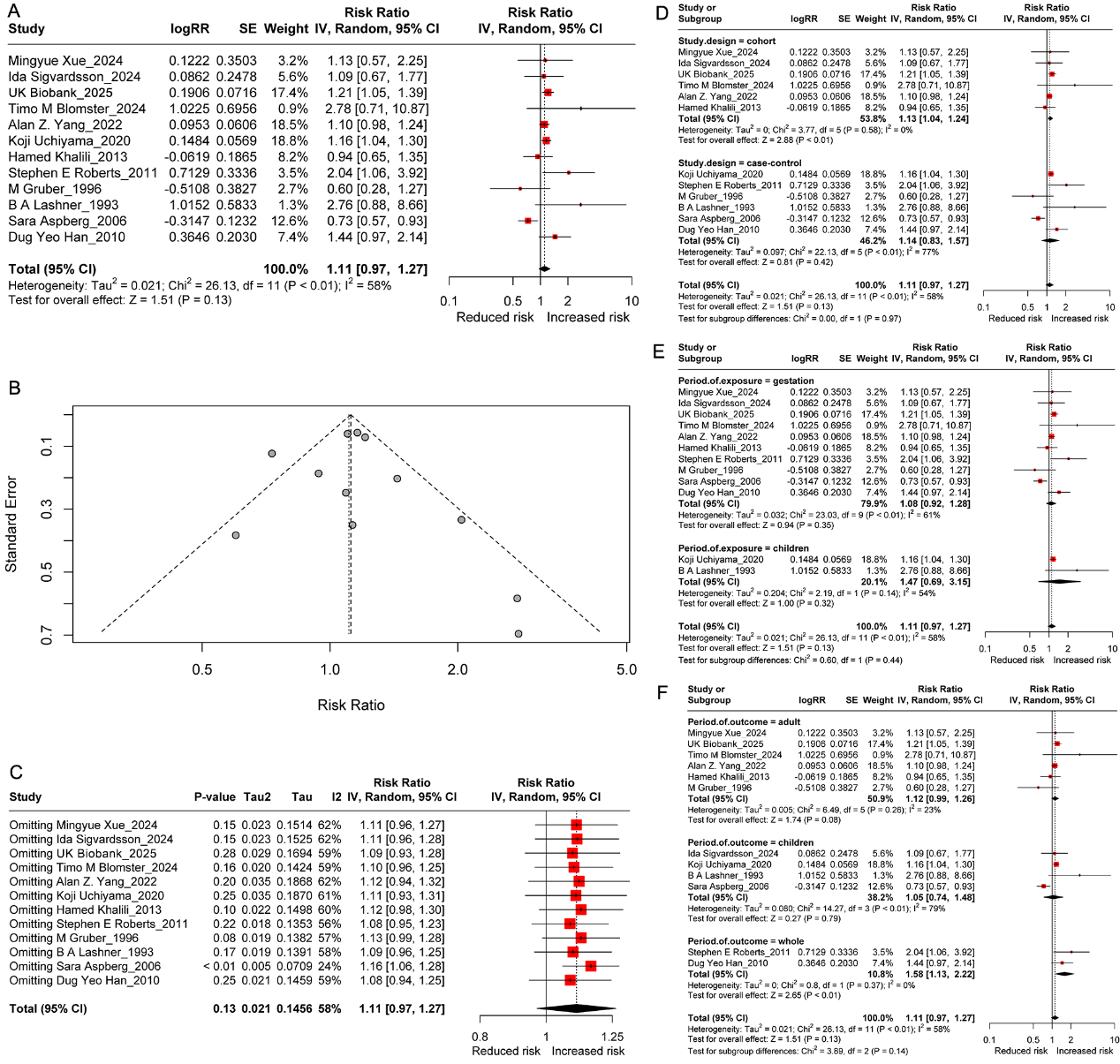


**eFigure 2. The results of meta-analysis of maternal smoking with the risk of Crohn’s disease in offspring.** A, forest plot. B, funnel plot. C, forest plot of trim and fill method. D, funnel plot of trim and fill method. E, forest plot of leave-one-out method. F, subgroup analysis according to the period of exposure. G, subgroup analysis according to the period of outcome. H, subgroup analysis according to the study design.Data are presented as odds ratios (ORs) with 95% confidence intervals (CIs) using the inverse-variance (IV), random-effects method. All tests were two-sided, and p < 0.05 was considered statistically significant.

**
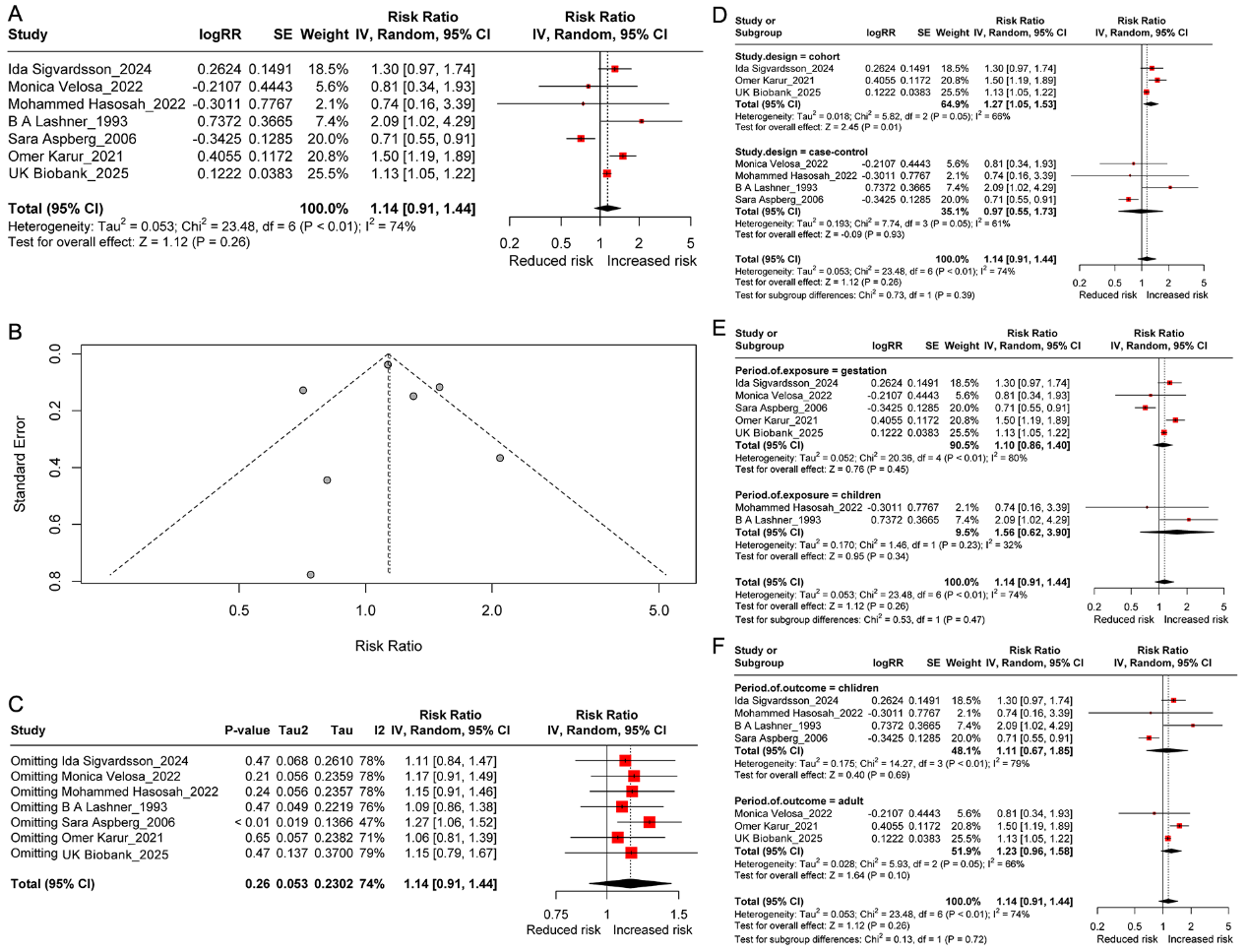
**

**eFigure 3. The results of meta-analysis of maternal smoking with the risk of overall inflammatory bowel disease in offspring.** A, forest plot. B, funnel plot. C, forest plot of trim and fill method. D, funnel plot of trim and fill method. E, forest plot of leave-one-out method. F, subgroup analysis according to the period of exposure. G, subgroup analysis according to the period of outcome. H, subgroup analysis according to the study design.Data are presented as odds ratios (ORs) with 95% confidence intervals (CIs) using the inverse-variance (IV), random-effects method. All tests were two-sided, and p < 0.05 was considered statistically significant.


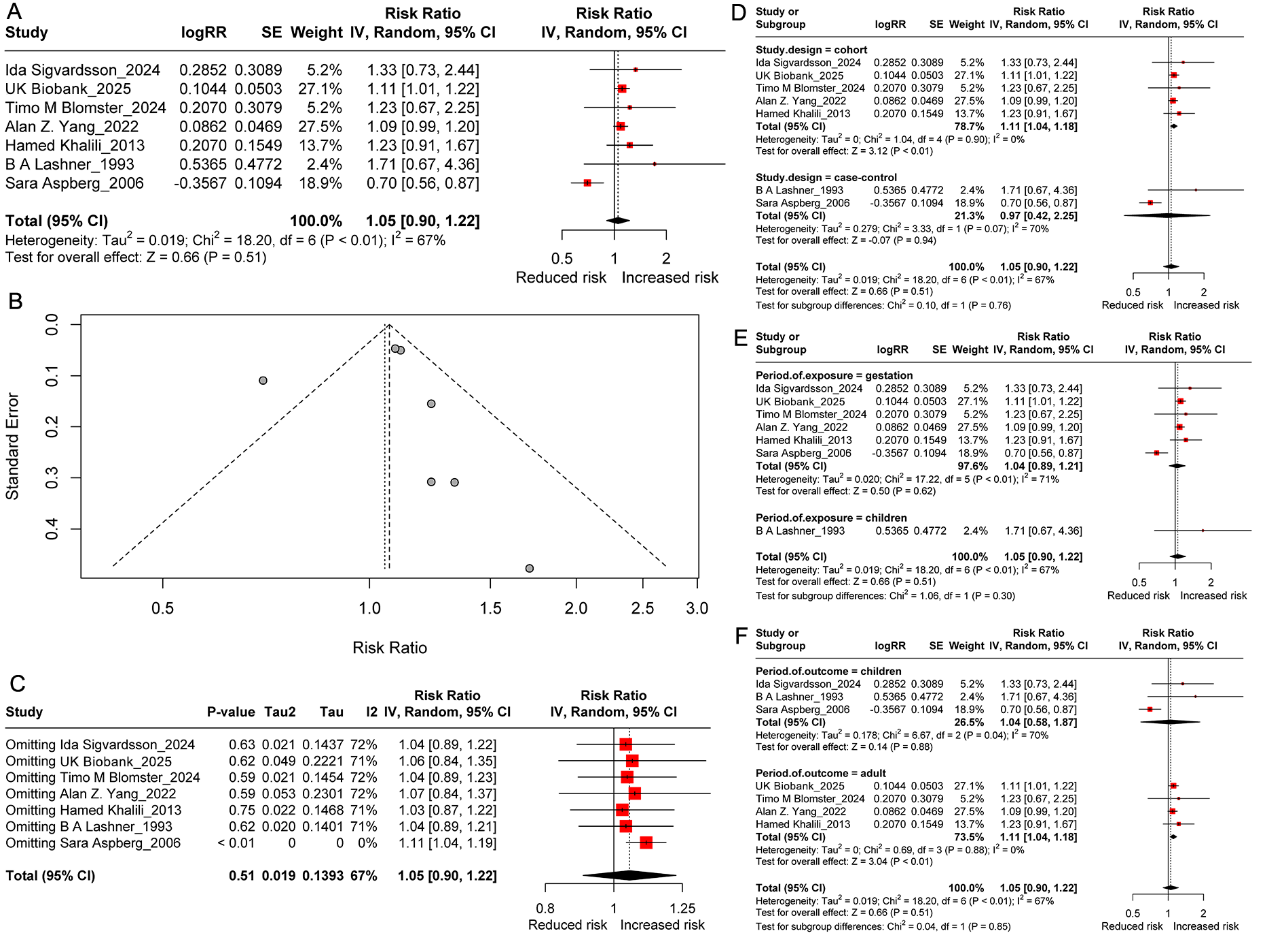


**eFigure 4. The results of meta-analysis of maternal smoking with the risk of ulcerative colitis in offspring.** A, forest plot. B, funnel plot. C, forest plot of trim and fill method. D, funnel plot of trim and fill method. E, forest plot of leave-one-out method. F, subgroup analysis according to the period of exposure. G, subgroup analysis according to the period of outcome. H, subgroup analysis according to the study design.Data are presented as odds ratios (ORs) with 95% confidence intervals (CIs) using the inverse-variance (IV), random-effects method. All tests were two-sided, and p < 0.05 was considered statistically significant.


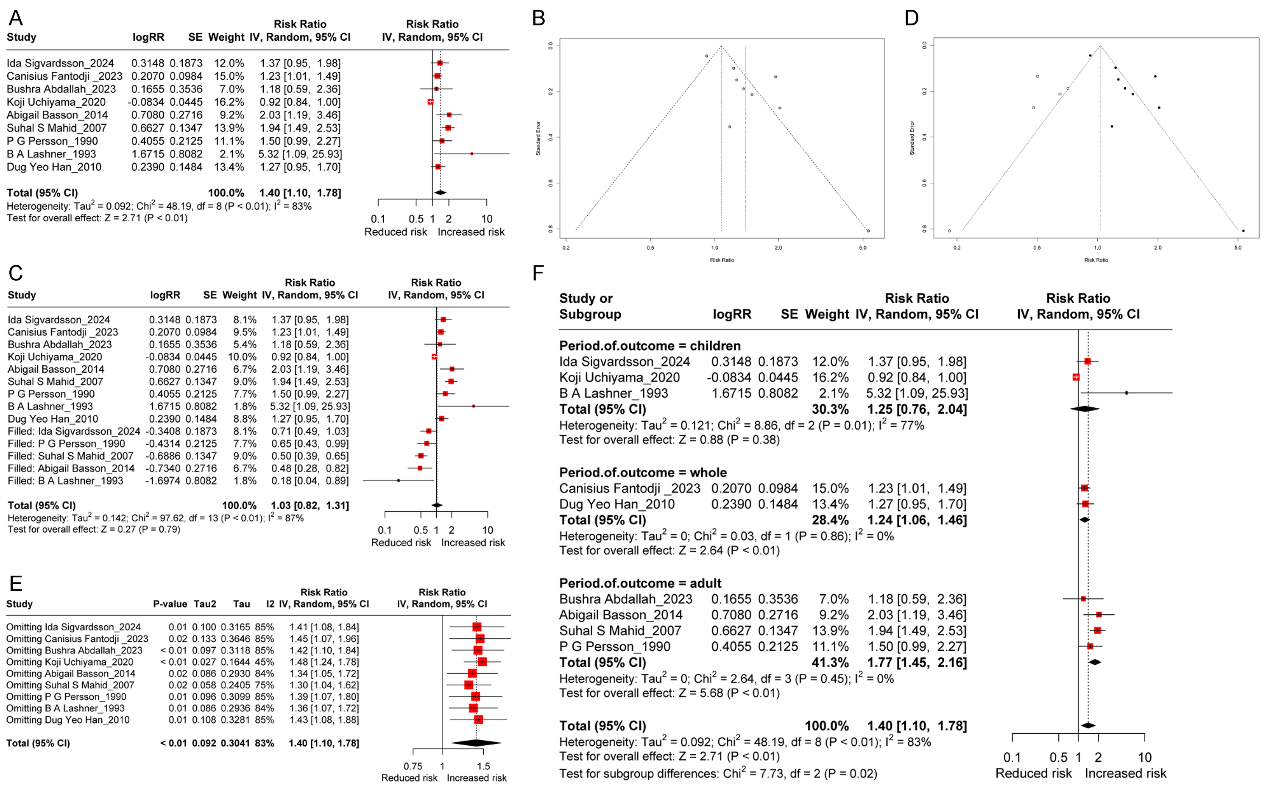


**eFigure 5. The results of meta-analysis of passive smoking exposure during childhood with the risk of Crohn’s disease.** A, forest plot. B, funnel plot. C, forest plot of trim and fill method. D, funnel plot of trim and fill method. E, forest plot of leave-one-out method. F, subgroup analysis according to the period of outcomeData are presented as odds ratios (ORs) with 95% confidence intervals (CIs) using the inverse-variance (IV), random-effects method. All tests were two-sided, and p < 0.05 was considered statistically significant..


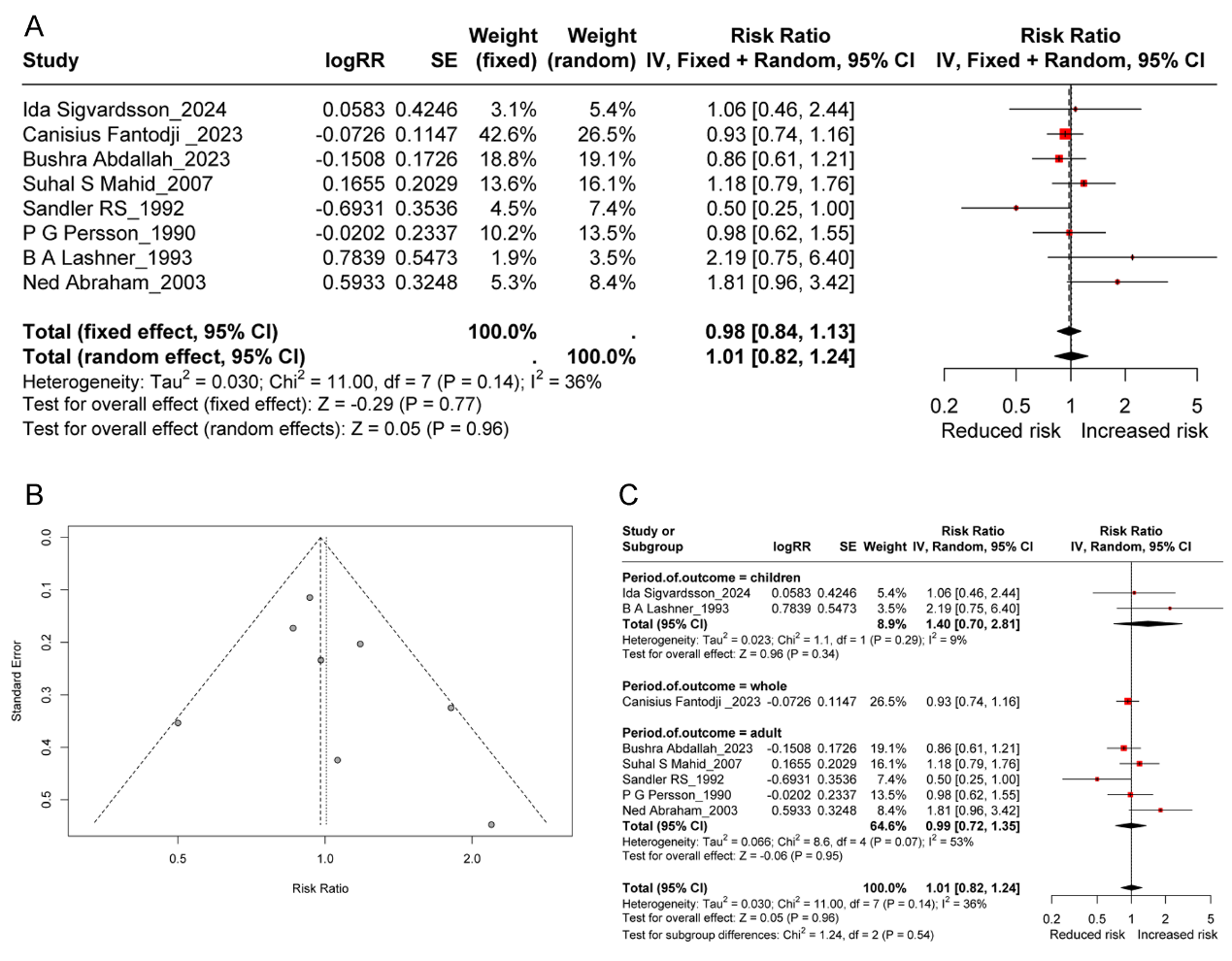


**eFigure 6. The results of meta-analysis of passive smoking exposure during childhood with the risk of ulcerative colitis.** A, forest plot. B, funnel plot. C, subgroup analysis according to the period of outcome.Data are presented as odds ratios (ORs) with 95% confidence intervals (CIs) using the inverse-variance (IV), random-effects method. All tests were two-sided, and p < 0.05 was considered statistically significant.

**
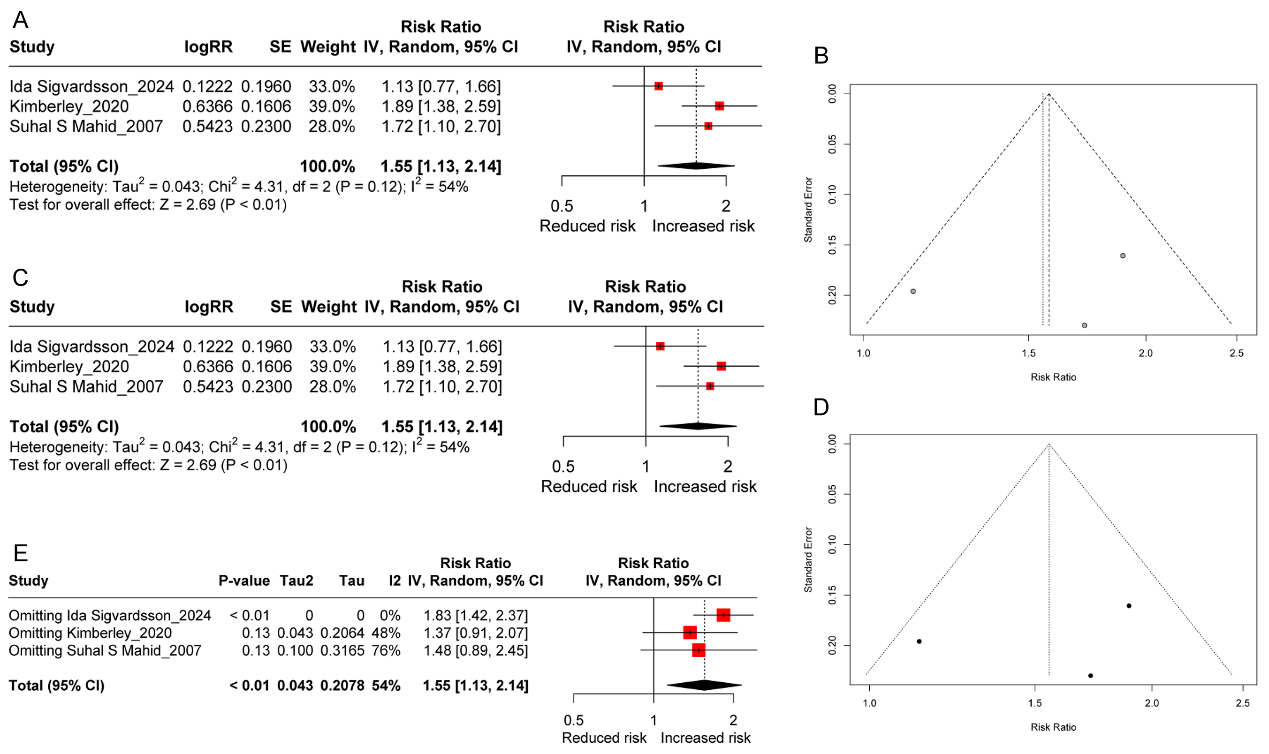
**

**eFigure 7. The results of meta-analysis of prenatal smoking exposure with the risk of Crohn’s disease in offspring.** A, forest plot. B, funnel plot. C, forest plot of trim and fill method. D, funnel plot of trim and fill method. E, forest plot of leave-one-out method.Data are presented as odds ratios (ORs) with 95% confidence intervals (CIs) using the inverse-variance (IV), random-effects method. All tests were two-sided, and p < 0.05 was considered statistically significant.


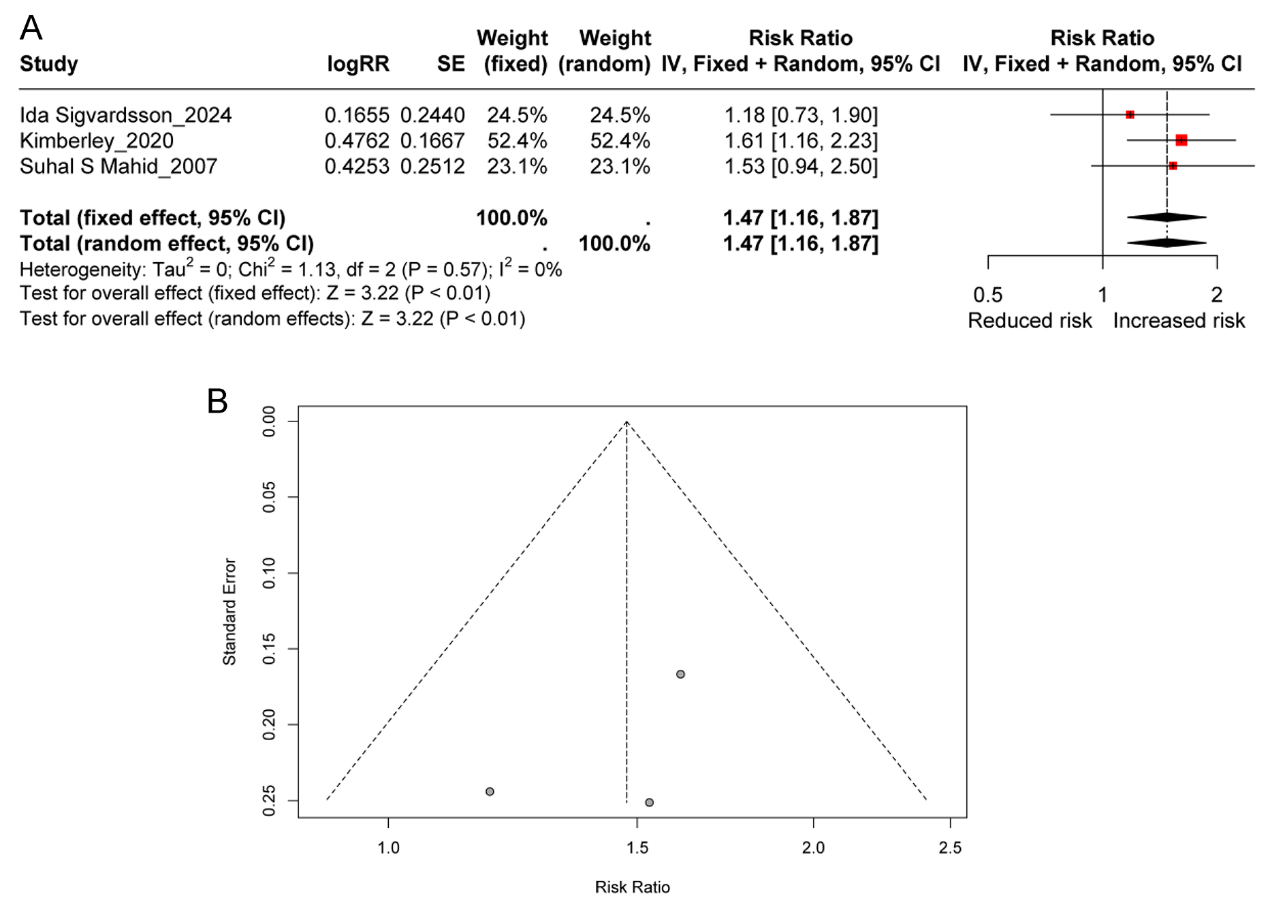


**eFigure 8. The results of meta-analysis of prenatal smoking exposure with the risk of ulcerative colitis in offspring.** A, forest plot. B, funnel plot. Data are presented as odds ratios (ORs) with 95% confidence intervals (CIs) using the inverse-variance (IV), random-effects method. All tests were two-sided, and p < 0.05 was considered statistically significant.


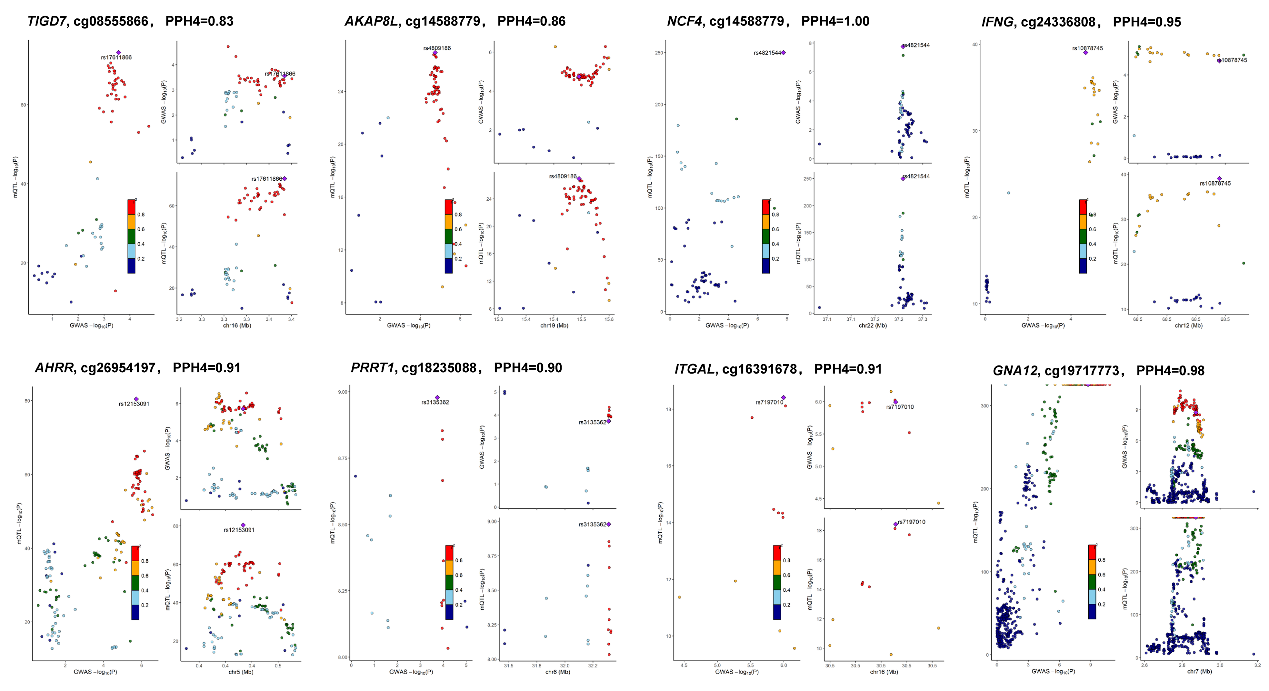


**eFigure 9. The region colocalization plot of those significant loci which identified in the epigenetic Mendelian randomization analysis.**


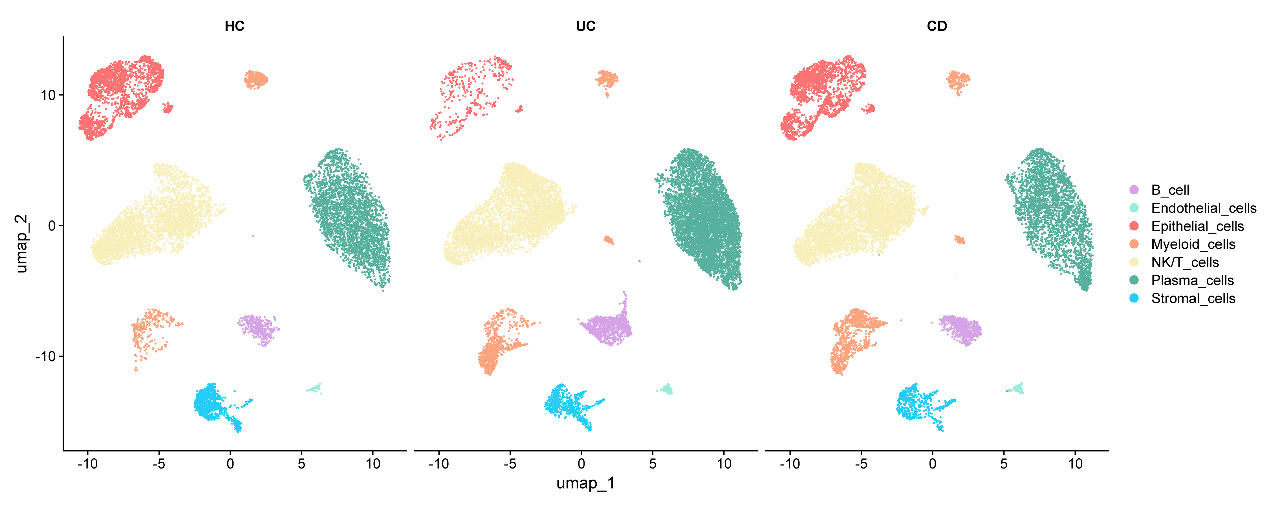


**eFigure 10. The annotation of cell types in intestinal of individuals with inflammatory bowel disease.**

**
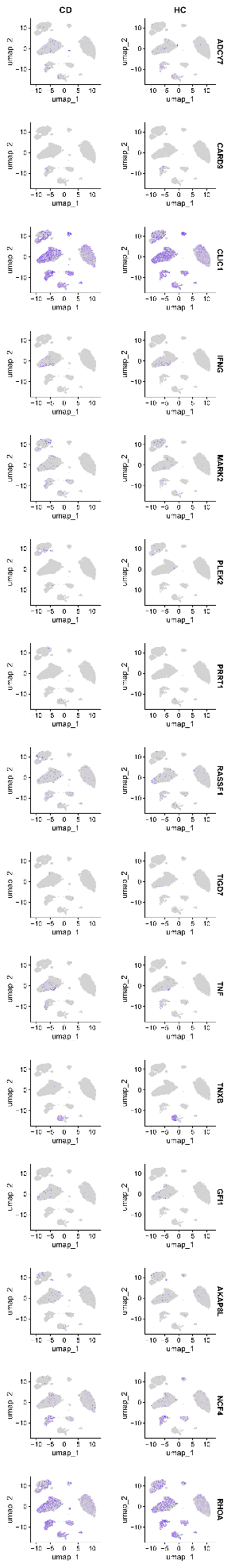
**

**eFigure 11. The global expression of significant loci in intestinal tissues of individuals with Crohn’s disease.**

**
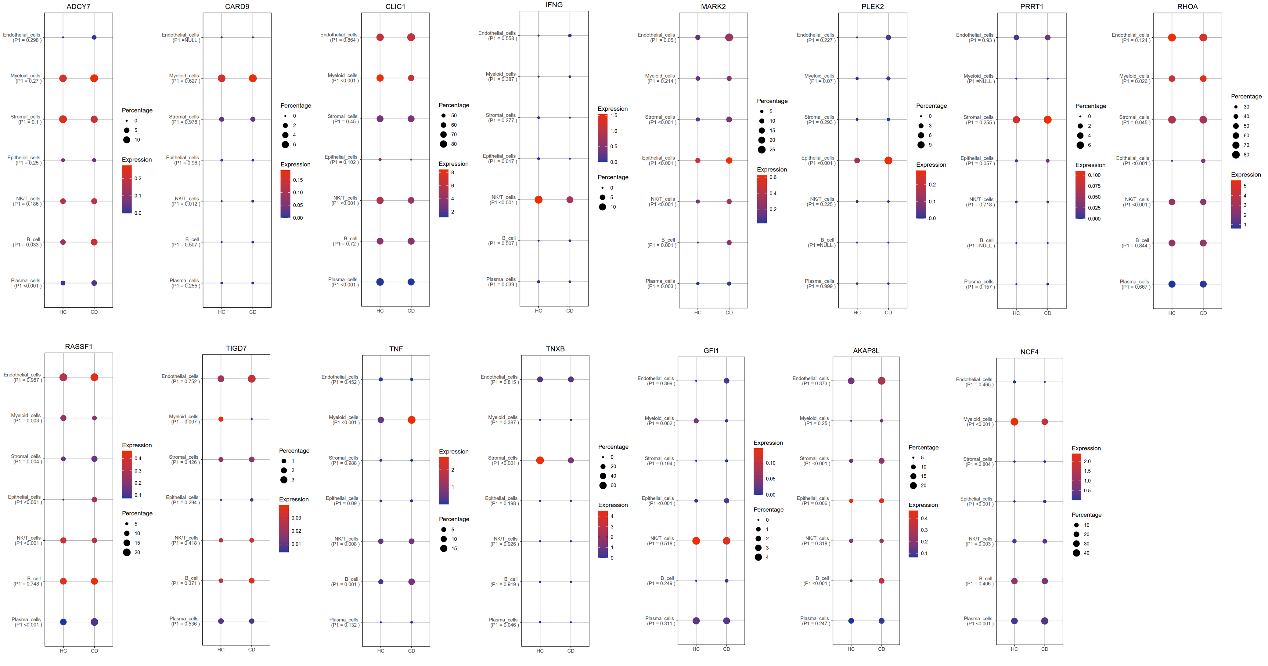
**

**eFigure 12. The expression of significant loci in specific intestinal cells of individuals with Crohn’s disease.**

**
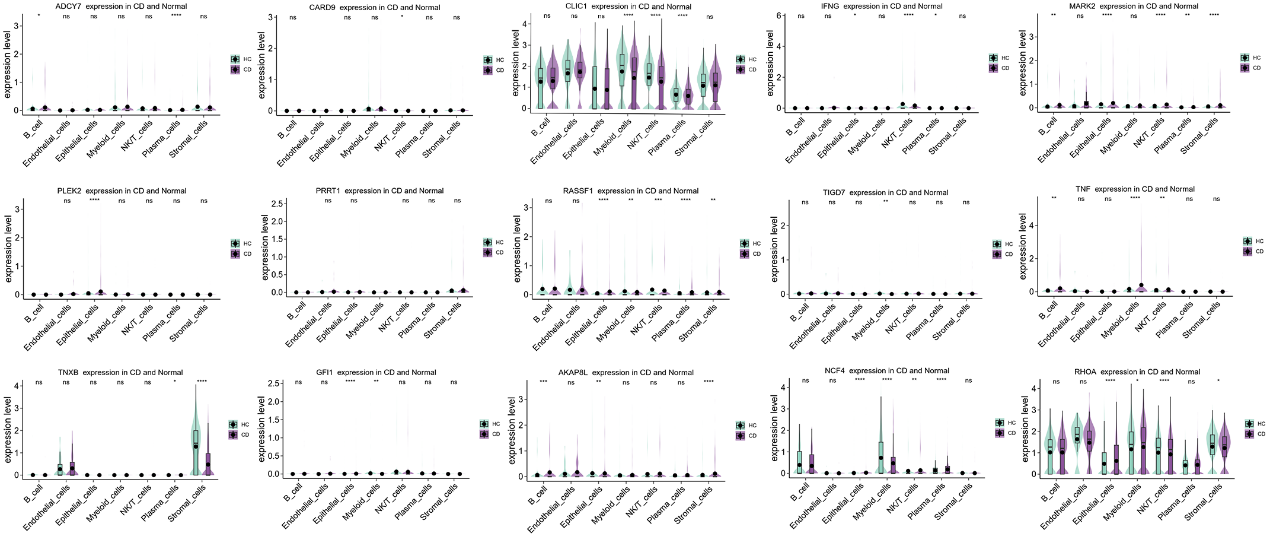
**

**eFigure 13. The percentage and expression differences of significant loci in specific cells of individuals with Crohn’s disease.**

**
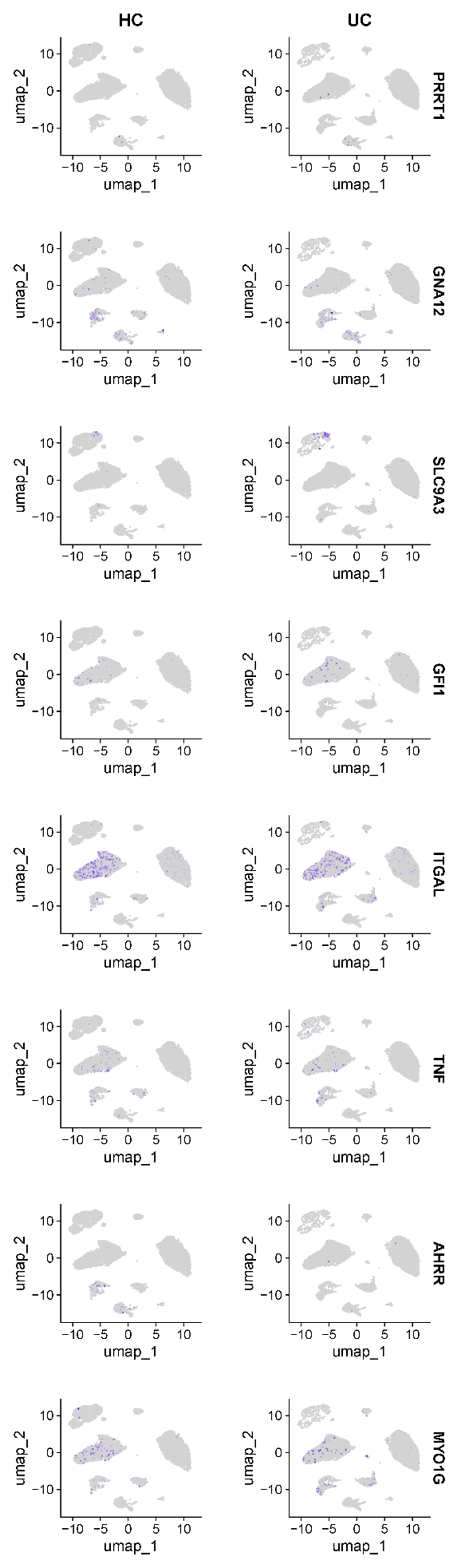
**

**eFigure 14. The global expression of significant loci in intestinal tissues of individuals with ulcerative colitis.**

**
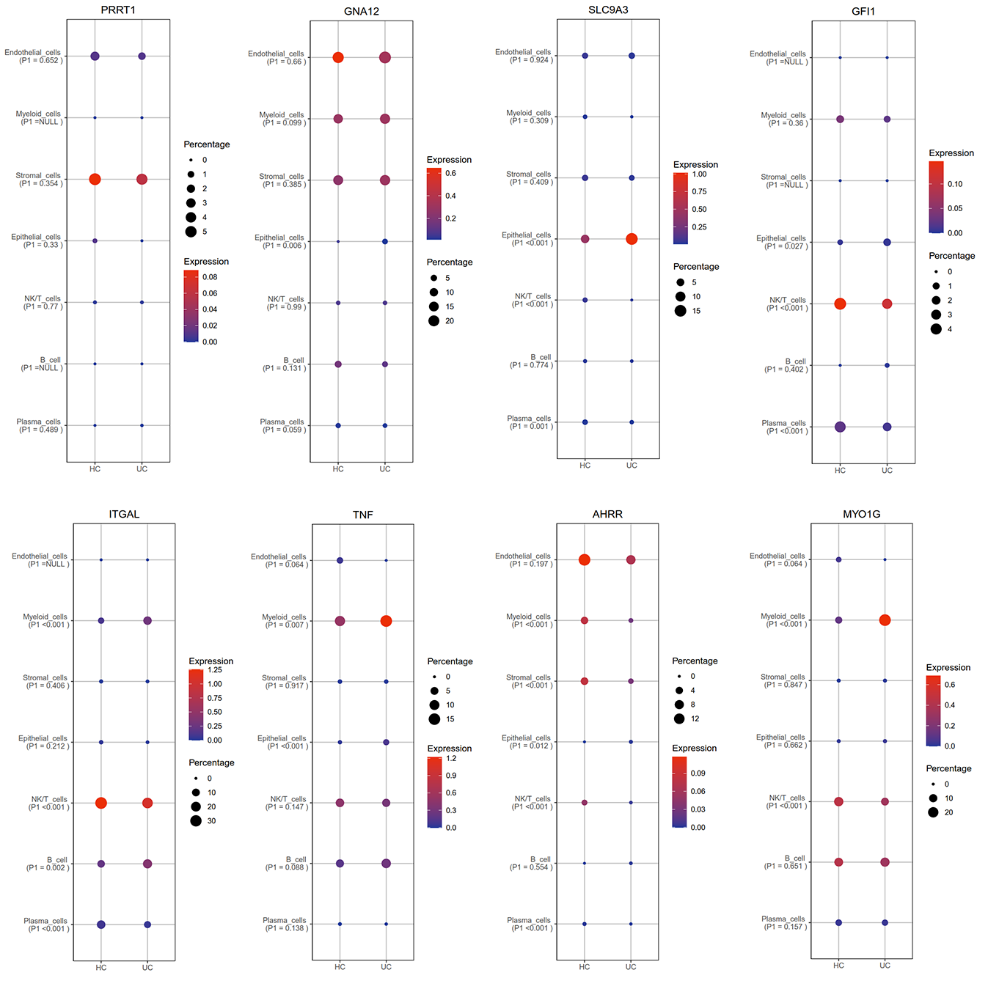
**

**eFigure 15. The expression of significant loci in specific intestinal cells of individuals with ulcerative colitis.**

**
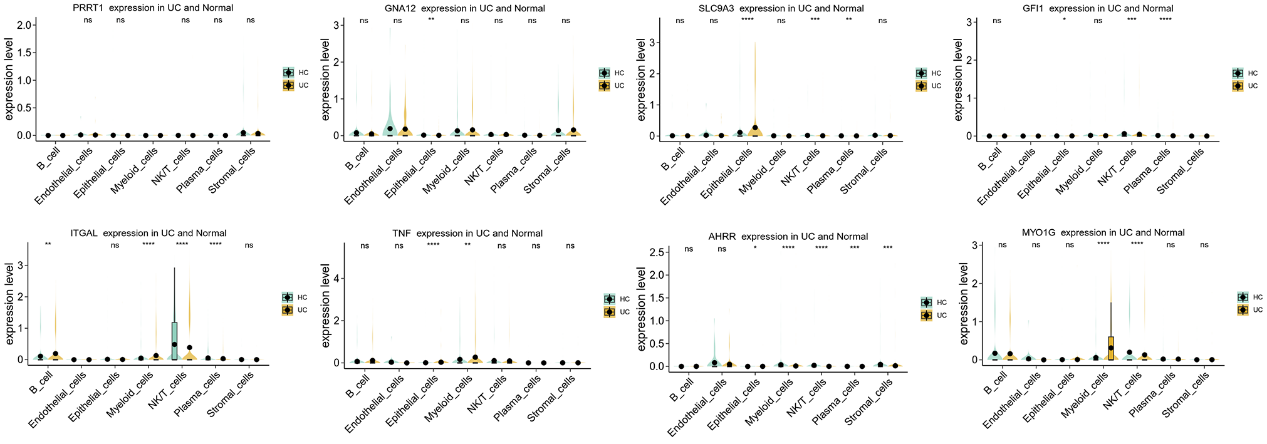
**

**eFigure 16. The percentage and expression differences of significant loci in specific cells of individuals with ulcerative colitis**

**eFigure 17. Gene expression profiles of different cell subpopulations from six CD patients compared with healthy controls.**

**
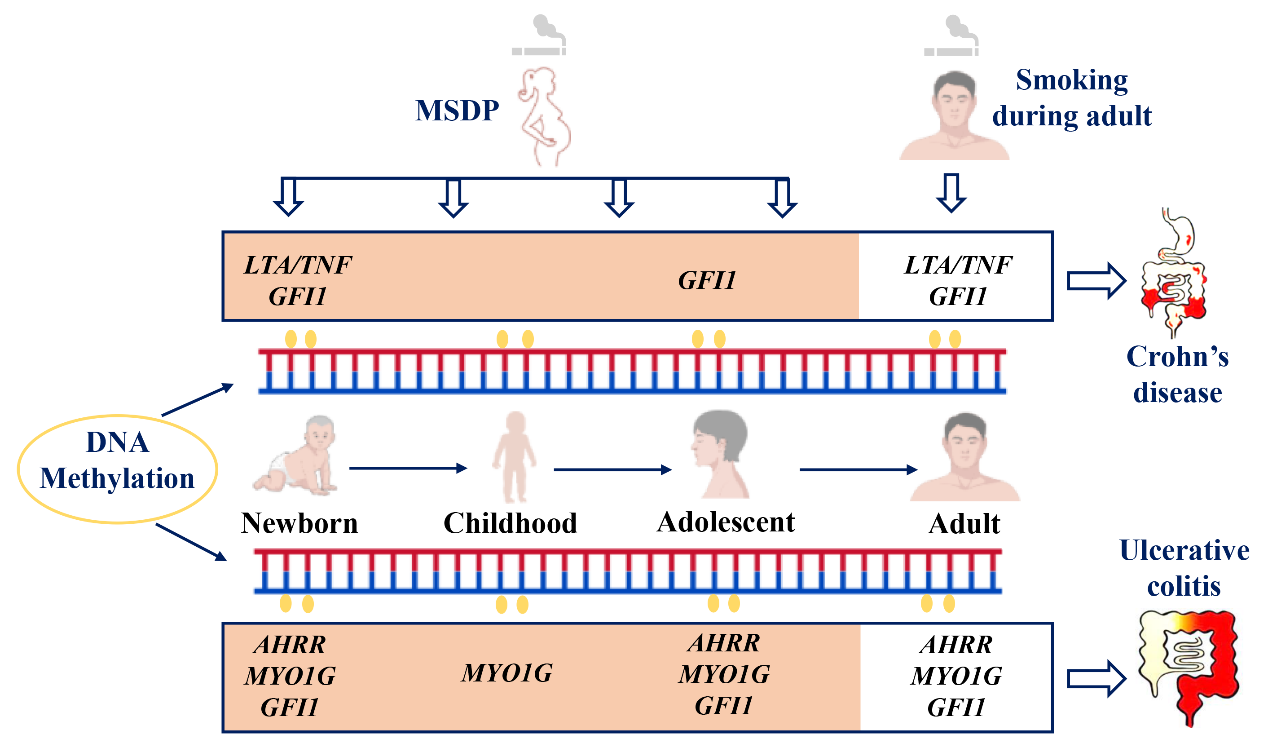
**

**eFigure 18. Shared DNA methylation sites related to smoking exposure at different life stages in inflammatory bowel disease**
